# Prioritization of indicators of the quality of care provided to older adults with frailty by key stakeholders from five canadian provinces

**DOI:** 10.1186/s12877-022-02843-9

**Published:** 2022-02-23

**Authors:** Anik Giguere, Jayna M. Holroyd-Leduc, Sharon E. Straus, Robin Urquhart, Véronique Turcotte, Pierre J. Durand, Alexis Turgeon

**Affiliations:** 1 Department of Family Medicine and Emergency Medicine, Université Laval, Quebec, Canada; 2 Departments of Medicine and CHS, University of Calgary, Calgary, Canada; 3 Department of Medicine, University of Toronto, Toronto, Canada; 4 Department of Community Health and Epidemiology, Dalhousie University, Halifax, Canada; 5 Quebec Excellence Centre on Aging, Quebec, Canada; 6 Department of Social and Preventive Medicine, Université Laval, Quebec, Canada; 7 Population Health and Optimal Health Practices Research Unit, Division of Critical Care Medicine, CHU de Quebec – Université Laval Research Centre, Université Laval, Quebec, Canada; 8 VITAM – Research Centre on Sustainable Health (Centre de recherche en santé durable), 2480, chemin de la Canardière, QC G1J 0A4 Québec, Canada

**Keywords:** Person-centered care, Quality improvements, Quality indicators, Patient outcome assessment

## Abstract

**Background:**

To meet the needs of older adults with frailty better, it is essential to understand which aspects of care are important from their perspective. We therefore sought to assess the importance of a set of quality indicators (QI) for monitoring outcomes in this population.

**Methods:**

In this mixed-method study, key stakeholders completed a survey on the importance of 36 QIs, and then explained their ratings in a semi-structured interview. Stakeholders included older adults with frailty and their caregivers, healthcare providers (HCPs), and healthcare administrators or policy/decision makers (DMs). We conducted descriptive statistical analyses of quantitative variables, and deductive thematic qualitative analyses of interview transcripts.

**Results:**

The 42 participants (8 older adults, 18 HCPs, and 16 DMs) rated six QIs as more important: increasing the patients’ quality of life; increasing healthcare staff skills; decreasing patients’ symptoms; decreasing family caregiver burden; increasing patients’ satisfaction with care; and increasing family doctor continuity of care.

**Conclusions:**

Key stakeholders prioritized QIs that focus on outcomes targeted to patients and caregivers, whereas the current healthcare systems generally focus on processes of care. Quality improvement initiatives should therefore take better account of aspects of care that are important for older adults with frailty, such as having a chance to express their individual goals of care, receiving quality communications from HCPs, or monitoring symptoms that they might not spontaneously describe. Our results point to the need for patient-centred care that is oriented toward quality of life for older adults with frailty.

**Supplementary Information:**

The online version contains supplementary material available at 10.1186/s12877-022-02843-9.

## Background

The demand for healthcare and support services will increase in the near future, as the population ages and becomes frailer [1]. In 2013, almost one-quarter of community-dwelling older adults in Canada are living with frailty, and about one-third with pre-frailty [2]. Frailty is defined as a dynamic state of vulnerability resulting from an aging-associated decline in one or more domains of human functioning (physical, psychological, social), which increases the risk of adverse outcomes and compromises the ability to cope with stressors [3]. Meeting the complex healthcare needs of a growing number of older adults with frailty will challenge our healthcare system in the coming years, as older adults with frailty are more likely to develop multimorbidity than their age-matched counterparts without frailty [1, 4, 5].

The current care of older adults with frailty is generally based on an acute care delivery model that is ill-suited to their complex care needs [6], resulting in suboptimal quality of care [7]. Indeed, older adults with frailty report difficulties accessing appropriate services [8], discontinuity of care and services [8, 9], and a general feeling of disempowerment in managing their own care [8–10]. Because older adults with frailty often experience multiple care transitions and navigate among numerous healthcare providers, they often experience medical escalation, redundant assessments [11], and avoidable visits to emergency departments [12]. The fragmentation of healthcare services and the challenges older adults with frailty experience accessing healthcare affect their quality of life [13].

Changes in the healthcare system are required, to better address the needs of the aging population [1]. Improving the integration of healthcare and social services has been proven to increase the quality of care provided to older people with frailty and their care experience, by reducing hospital usage [14–19], family/friend caregiver burden [15], overall care costs [15, 16, 19], and by limiting delays in care transfer by providing continuity [15]. Moreover, integrated home care and services have been shown to facilitate end-of-life care at home, often preferred by patients [18, 20].

To develop and evaluate care models that meet the needs and priorities of older people with frailty more effectively, it appears essential to understand which aspects of care older people feel are suboptimal [21]. It is therefore important to use patient-centred indicators such as Patient-Reported Outcome Measures (PROMs) and Patient-Reported Experience Measures (PREMs) [22, 23].

Using indicators of the quality of care specific to older adults with frailty, and coherent with their needs, would help optimize patient safety and align data-driven approaches with patient-centred care. In practice, specifying a benchmark for each quality indicator, and using audit and feedback mechanisms could effectively support quality improvement, by informing healthcare professionals and organizations about their performance compared to peers [24, 25]. In principle, healthcare professionals and organizations would be prompted to modify their practices if performance feedback show that they are inconsistent with a desirable target, and if they have the resources to modify them [24].

Therefore, our study sought to identify and prioritize quality indicators (QIs) for monitoring outcomes among older adults with frailty. The specific research questions addressed were: [1] How do key stakeholders rate the importance of QIs to monitor the health of older adults with frailty? and [2] How do they explain their ratings?

## Methods

This was a convergent mixed-method study comprising quantitative data collection in the form of a survey, as well as qualitative data collection via individual interviews. The study was part of a larger project that aimed to examine the care of older adults with frailty across a spectrum of settings in five Canadian provinces. The larger project also comprised a scoping review of the literature, a qualitative study to explore the views of key stakeholders regarding the quality of care of older adults with frailty [26], and a multivariate analysis of administrative data [27, 28].

### Study participants

 We recruited a convenience sample of healthcare providers (HCPs) involved in the care of older adults with frailty, together with healthcare administrators or policy/decision makers (DMs) involved in decisions regarding the care of older adults with frailty, via the networks of research team members in five Canadian provinces (Quebec, Ontario, Nova Scotia, Alberta, British Columbia). We also recruited a convenience sample of people who were 65 years of age or older and were considered to be experiencing frailty, as judged by their HCPs based on two widely used scales, the Clinical Frailty Scale [29] or the Edmonton Frail Scale [30]. Older adults with both frailty and cognitive impairments were eligible to participate, provided their family caregiver accompanied them. Family caregivers (broadly defined as family and/or friends involved in the care of an older adult) assisting older adults with activities of daily living were also eligible. We used two recruitment strategies. First, we asked the participating HCPs to identify potential participants among their patients and provide them a study information sheet inviting them to contact the research team if they wished to participate. Second, we placed posters in geriatric medicine clinics inviting potential participants to contact the research team if they were interested in participating.

### List of clinical quality indicators

In keeping with the Agency for Healthcare Research and Quality’s (AHRQ) taxonomy of quality measures [31], we developed a list of 36 clinical quality indicators (QIs) through a scoping review of the literature (described in the Additional file 1) and an expert panel consultation (described in the Additional file 2).

### Survey of clinical quality indicators

We asked participants their level of agreement with the importance of each of these 36 indicators of quality of care in a self-reported survey, using 5-point Likert scales ranging from 1 (strongly disagree) to 5 (strongly agree). We developed a tailored version of the survey for patients and family caregivers by adjusting its reading level to below grade-9 using a readability test tool [32] and plain language techniques to ensure the indicators were easy to read and to understand. This version of the survey was pilot-tested with two potential users and adapted to improve understanding prior to its use with study participants.

### Interview procedure

From June to October 2015, six members of the research team conducted in-depth interviews, adhering to a semi-structured guide to explore participants’ motivations for how they agreed with the QIs. We tailored the interview guide for patients and family caregivers, and used probes to help participants clarify and elaborate on their views. We conducted the interviews with HCPs and DMs over the phone, and those with older adults and/or their family caregivers in person. Interviews lasted approximately 50 min, and were audio recorded and professionally transcribed. One participant refused to be recorded but agreed to note-taking as an alternative.

### Data analyses

We conducted descriptive statistical analyses on all the studied variables. We also conducted an exploratory analysis to compare the frequency distributions of participants’ ratings of indicators stratified by the type of participant (older adults and family caregivers, HCPs, and DMs), using a Fisher’s exact test. The analysis was performed using SAS version 9.4 (SAS Institute Inc., Cary, NC, USA), with an alpha level of 0.05.

We used a deductive approach in the qualitative analysis of interview data. The analysis applied the AHRQ taxonomy of quality measures [31], and also allowed for the inclusion of new themes that emerged from the interview data. Data were entered into a qualitative data analysis software (NVivo version 10, QSR International). Three research team members (of whom AG), worked on the analysis to carry out the coding, corroborating it and verifying the consistency between the themes and the interview content, before validating with the research team.

## Results

### Study participants

 We recruited forty-two participants: eight older adults with frailty and their family caregivers, 18 HCPs, and 16 DMs. Tables 1 and 2 present the participants’ characteristics. The number of participants varied across provinces. In QC and BC, we recruited frail seniors or their caregivers through participating HCPs. In the other provinces where we used posters in geriatric clinics, recruitment of frails seniors or their caregivers remained unsuccessful despite all efforts. 61% of HCPs were physicians, and more than half of them were specialized in geriatric care. Seven of the 18 HCPs recruited worked in primary care settings. 62% of DMs worked in provincial healthcare systems.

**Table 1:** Socio-demographic and professional characteristics of participants (*DM* = Decision Maker, *HCP* = Healthcare Professional)

Characteristic	DM (*n*=16)	HCP (*n*=18)	Patient (*n*=5)	Caregiver (*n*=3)
**Gender**				
Female	13	11	1	2
Male	3	7	4	1
**Age (years)**				
25-34		1		
35-44	1	5		
45-59	15	6		2
60-64		4		
65+		2		
65-74			1	
75-84			2	
85+			2	
NA				1
**Province of Canada**				
Alberta (AB)	4	4		
British Columbia (BC)	4	4	3	1
Nova Scotia (NS)	2	2		
Ontario (ON)	1	4		
Quebec (QC)	5	4	2	2

**Table 2:** Professional characteristics of participating (**A**) Decision Makers (DMs), and (**B**) Healthcare Professionals (HCPs)

A- Decision makers (DMs) characteristics	Frequency (*n*=16)
**Management experience (years)**	
6-10	4
11-15	2
16-20	2
21-25	5
26-30	2
31-35	1
**Type of organization**	
Provincial health system	10
University	1
Hospital	1
Advocate for older adults	1
Medical Association	2
Regional Health Agency	1
**Level of organization**	
Regional	3
Provincial	11
National	2
**Role in the organization**	
Operations	5
Planning	1
Operations, planning and finance	2
Other	8
**Educational background**	
MSc related to administration (e.g. MBA, Public administration)	7
MD	6
Nurse	3
**B- Healthcare professionals (HCPs) characteristics**	**Frequency (*n*=18)**
**Profession**	
Physician	11
Nurse	2
Social Worker	2
Other	3
**Specialization in geriatric care**	
Yes	11
No	7
**Practice experience (years)**	
1 -10	5
11-20	3
21-30	5
31-40	4
N/A	1
**Language used at work**	
English	14
French	4

### Survey Results

Participants’ ratings resulted in six of the 36 QIs listed in the survey being more important: (1) increasing the quality of life of patients; (2) increasing healthcare staff skills; (3) decreasing patients’ symptoms; (4) decreasing family caregiver burden; (5) increasing patient satisfaction with care; and (6) increasing family doctor continuity of care (Table 3).

**Table 3:** Participants’ level of agreement with the value of QI of care provided to older adults with frailty

Quality Indicator (QI)	Level of agreement ranging from 1 (strongly disagree) to 5 (strongly agree)	
	**Mean (SD)**	**Median (min; max)**
1) Increase in quality of life of the patient	4.7 (0.5)	5 (3; 5)
2) Increase in healthcare staff skills	4.5 (1.6)	5 (3; 5)
3) Decrease in symptoms	4.5 (1.7)	5 (2; 5)
4) Decrease in caregiver’s burden (psychological, physical, or financial costs experienced by a caregiver providing homecare to a older adult with frailty)	4.4 (1.7)	5 (2; 5)
5) Increase in patient satisfaction with care	4.4 (1.7)	5 (3; 5)
6) Increase in family doctor continuity of care over the last year of life	4.4 (1.7)	5 (3; 5)
7) Decrease in the rate of o who have experienced non-beneficial medical care during their last year of life (ventilation, resuscitation, operating room/surgery)	4.3 (1.7)	5 (2; 5)
8) Decrease in the rate of hospital readmission	4.3 (1.7)	4 (1; 5)
9) Decrease in risk of falling	4.3 (1.7)	4 (2; 5)
10) Decrease in the rate of visits to the emergency department	4.2 (1.7)*	4 (2; 5)
11) Increase in healthcare staff knowledge	4.2 (1.6)*	4 (3; 5)
12) Increase in the ability of patient to cope with difficulties, changes, and emotional struggles that arise with aging (coping effectiveness)	4.2 (1.7)	4 (2; 5)
13) Increase in patient empowerment (becoming self-sufficient)	4.2 (1.7)	4 (2; 5)
14) Decrease in unmet needs of the patient	4.2 (1.7)	4 (2; 5)
15) Increase in physical capacity (gait, balance)	4.2 (1.7)*	4 (2; 5)
16) Decrease in depression (having the blues)	4.2 (1.7)	4 (2; 5)
17) Decrease in the number of hospital days during last year of life	4.2 (1.8)*	4 (1; 5)
18) Increase in healthcare staff’s respect of best practices	4.2 (1.8)	4 (2; 5)
19) Decrease in the number of intensive care unit admissions during last year of life	4.1 (1.9)	4,5 (1; 5)
20) Decrease in the number of new hospital admissions during last year of life	4.1 (1.8)	4 (1; 5)
21) Decrease in patient helplessness (feeling of being powerless)	4.1 (1.7)	4 (3; 5)
22) Decrease in the use of acute inpatient hospital services, such as receiving short-term treatment for a severe injury or episode of illness, an urgent medical condition, or during recovery from surgery	4.1 (1.8)	4 (2; 5)
23) Decrease in the rate of falls	4.1 (1.8)	4 (2; 5)
24) Increase in patient independence (autonomy)	4.0 (2.0)	4 (2; 5)
25) Decrease in social isolation of the patient	4.0 (2.0)	4 (3; 5)
26) Decrease in the number of placements in long-term care/nursing homes	4 (1.8)	4 (2; 5)
27) Increase in the rate of older adults with frailty who receive care from a palliative care organization	3.9 (1.7)	4 (2; 5)
28) Increase in nutritional status	3.9 (1.7)	4 (2; 5)
29) Location where the older adult with frailty spent the majority of their time during last year of life	3.97 (1.8)	4 (2; 5)
30) Increase in multidisciplinary care: rate of family doctor visits over all visits made at clinics during the last year of life	3.892 (1.8)	4 (2; 5)
31) Increase in the rate of family doctor visits over all doctor visits during the last year of life	3.7 (1.8)	4 (2; 5)
32) Increase in mental function (cognitive performance)	3.7 (1.9)*	4 (1; 5)
33) Receiving at least one physician house call during last year of life	3.6 (1.7)	4 (2; 5)
34) Increase in the number of family doctor visits during last year of life	3.4 (1.8)	3 (2; 5)
35) Decrease in the number of visits to specialists at a clinic during last year of life	3.3 (1.8)	3 (2; 5)
36) Decrease in risk of death	3.1 (1.8)	3 (1; 5)

*Indicates significant differences in perceived value between patients, healthcare professionals, and decision makers

The ratings of five other QIs, notably the number of inpatient days during the last year of life, rate of emergency department visits, physical capacity (gait, balance), provider knowledge, and cognitive performance varied by type of participant. Specifically, a post hoc analysis showed that older adults placed less value on the number of inpatient days during the last year of life, but valued provider knowledge more compared to HCPs and DMs. Variations were not statistically significant for the rate of emergency department visits, physical capacity, and cognitive performance (see Additional file 3).  The participants provided a written informed consent to participate. 

#### Participants’ motivation for the ratings

In the interviews, participants explained their motivation for assigning their ratings of top-rated indicators. The majority emphasized the importance of improving patients’ quality of life, and considered this indicator as far more important than, for example, decreasing risk of death. Participants shared a strong feeling that older adults with frailty must be able to define their quality of life, because that allows them to define their own care goals and meet them accordingly. Overall, all participants viewed this indicator as essential to raise awareness about older adults’ perspectives in the process of care. Participants also suggested that quality of life should be measured and reported along with other patient-reported outcomes.

*I think they all ultimately play into quality of life and what the patient and their loved ones, their caregivers, feel that’s important… what’s important to them. (Alberta, DM#3)*

Healthcare providers’ skills were rated close behind quality of life. Participants shared concerns that poor or deficient skills may limit the quality of care and services older adults with frailty receive, as well as their quality of life. Participants also discussed what the term “skills” included, and suggested that awareness and support in providing assistance with activities of daily living should be considered an essential skill.

*An increase in the provider’s competency or skills, that’s another side of the equation, but if you are looking strictly at the quality of a clinical intervention, certainly, increasing the knowledge and skills of the people delivering that clinical intervention should result in a strong increase in quality. (Alberta, DM#4)*

Participants considered it important to decrease symptoms since they can influence patient autonomy and quality of life. They expressed concern that symptoms, especially pain, often remain undetected by HCPs and are underreported by patients. Therefore, participants suggested that symptoms must be assessed thoroughly as part of a geriatric assessment.

*I think reduction of symptoms, certainly. That is going to be an indicator of better quality of life in most instances, again, if I think of palliative care patients. If the symptoms are better managed, there is usually a better quality of life […]. (British Columbia, HCP #4)*

Participants also prioritized the need to decrease the burden on family caregivers. According to them, family caregivers are overworked and need support, however, the issue remains largely unaddressed at this time. Participants stated that a sustainable healthcare system relies on family caregivers to help keep older adults with frailty at home, manage their quality of life, and avoid institutionalization and, therefore, caregiver burden must be measured and reported.

*If we don’t understand how to reduce caregiver burden, we’re not going to have a sustainable system. So that is really, really, really important, I think, from everyone’s perspective. (Alberta, DM #2)*

Participants explained that older adults with frailty and HCPs might not have the same views about patient satisfaction regarding care, and that this indicator helps obtain patients’ views on several other important indicators, such as quality of life, caregiver burden, or patient coping effectiveness and autonomy. Consequently, some suggested that “experience with care” would be a more appropriate indicator than “patient satisfaction”. Although some expressed concerns with patient satisfaction being a subjective measure, the majority valued this indicator because it provides insight into older adults’ views and experiences with care.

*For me, “patient satisfaction with care,” I would maybe reframe that to “satisfaction with their experience with the system of care,” because some older adults will say, if it’s the care which is actually the nurse at the bedside or the care in the home, yes they are satisfied … but surrounding that care is a system with which they’ve had great frustration. (British Columbia, DM #4)*

Participants viewed continuity of care provided by a family doctor over the last year of life of the older adult with frailty as important because family doctors know their patients well, they are there for older adults, and are perceived by participants as partners in the care of older adults with frailty.

*I think that the family physician piece [of the puzzle] is important, particularly for those who have long-standing relationships with their family doctors. […] They often know the individuals very well and I think the more we can tap into that, the more we can often make a difference in the quality of care. (British Columbia, HCP #4)*

With the exception of continuity of care, participants perceived that practical measurement of the highest-rated indicators may not be feasible.

## Discussion

Our study sought to identify important QIs for monitoring outcomes among older people with frailty. A scoping review and expert consensus allowed to list 36 QIs, which were then prioritized by stakeholders using first a survey and then semi-structured interviews. The ratings and the interview findings shed light on a number of interesting findings, discussed hereafter.

The proposed list of QIs is extensive and concurs with several of the QIs proposed in another such list put together to evaluate geriatric-led care models [33]. However, our list of QIs and that of this other study are both based on the scientific literature and the opinion of expert clinicians, researchers or health managers; they therefore do not directly include the perspectives of older adults living with frailty and their caregivers. A next step will therefore require to validate these lists and complement it through a patient-centered prioritization process, such as that used by the James Lind Alliance for setting research priorities [34].

Study participants rated increase quality of life as the most important indicator of the quality of care for older adults with frailty. They also expressed that older people with frailty should define for themselves what could have an impact on their quality of life, and set their own goals of care. Current healthcare systems generally focus on inputs to and processes of care [22], without always considering quality of life and other patient-reported outcomes measures as objectives of care. Individualized measures, such as the Goal Attainment Scale [35] or the Canadian Occupational Therapy Performance [36], are promising strategies to better evaluate care according to older adults’ own goals, as they help quantify their progress in relation to the goals they set themselves. The implementation of such measures could help address the substantive gaps in the assessment of healthcare quality and outcomes from the perspective of the patients themselves and their family caregivers.

Beyond making quality of life a priority, participants in the current study also reported patient satisfaction with care as a relatively important quality indicator for older adults. Consistent evidence suggests that the most important health service factor affecting satisfaction is the patient–practitioner relationship, including information giving [37]. On the other hand, the struggle to exercise control over their decisions and to maintain their sense of personal value can limit older adults’ satisfaction with care [9]. Hence, to offer quality and satisfactory care to older adults, it appears essential to develop HCP’s communication skills, and to train them better in the provision of person-centred care focussed on the older adult’s needs and priorities, and in shared decision-making [10].

Participants also valued continuity of care from the family doctor (indicator #6) over having access to specialists (indicator #35). They explained that they valued long-standing relationships between patients and family doctors, since such relationships allow patients to be well known and enable their involvement as a care partner. This is consistent with other studies that report the importance older adults place in continuity of primary care [38]. Several studies report how continuity of primary care is associated with improved health outcomes among the older population, including lower rates of potentially inappropriate medication prescription [39]. Additionally, according to previous studies [40, 41], sustained continuity of care is also associated with reduced hospitalizations (indicator #17) and unnecessary emergency room visits (indicator #10), two items also rated as relatively important in the current project. The study participants appreciated that measuring continuity of care is feasible; it can indeed be assessed through questionnaires [42, 43]. Continuity of care of older adults with frailty can also be assessed using administrative databases [44], based on the Modified Modified Continuity Index [45]. However, even if continuity can be effectively measured, the challenge as to the most effective strategies to improve it remains. Key quality improvement strategies to improve continuity of the care of people living frailty include increased professional training on quality assurance, more resources in primary care to support the extra documentation required, a better access to allied health professionals, and standardized electronic medical records across settings [25].

We also noted that the study participants valued HCPs' skills in older adult care as an indicator of care quality. In the interviews, they expressed some concerns regarding the current level of HCPs' skills, which may explain why they perceived this QI as important. They explained that these skills largely determine the quality of care and services provided to older adults with frailty and, in turn, their quality of life-thus linking back to the most prominent indicator identified in this work. Providing future HCPs with quality experiences in caring for and interacting with older people has the potential to increase positive attitudes toward the care of older adults [46, 47].

In addition, participants valued the monitoring of symptoms to assess care quality for older adults; the value they placed on this indicator reflected their views with frailty that some symptoms often remain undetected by HCPs and underreported by patients. Prior studies indeed showed that depression [48] and pain [49] were commonly hidden or underreported among older people, who often believed they were part of the normal process of aging [49]. Hence, there is a need to educate older adults on the importance of describing their symptoms and to not be too quick to dismiss symptoms as part of normal aging.

Participants in the current study also rated the need to decrease the burden on family caregivers as important, to avoid them from developing disabilities themselves. As family and friends represent a key resource for the care and quality of life of older adults with frailty [10, 50], it is essential to better prepare future HCPs to meet their needs as well as those of the patient, and to consider them as partners in care with frailty [50].

Our exploratory analyzes achieved with a limited sample size suggests that patients could have different priorities than other types of participants. For instance, the indicator concerning the number of days spent in the emergency room was rated as more important by managers and health professionals than it was by patients. Such disparity should be studied further considering that reducing the number of emergency department visits is often reported as quality indicator of end-of-life care [51]. Quality improvement research initiatives that target care indicators that are important to patients have started to take place in Canada [52], and elsewhere [53] to address these issues.

Finally, it is noteworthy that the indicator ranked as least important to participants among the 36 proposed was “decrease in risk of death”. Participants clearly placed more importance on quality of life than on risk of dying. Vulnerable older adults, regardless of their cultural or religious background, do not want to live at all costs if it means that their quality of life is compromised [54].

### Study limitations

 Our study recruited a convenience sample of participants, and our sample size was small. The participants who agreed to participate may thus be different from the general population. This might not have reflected the true diversity of experiences and views in the field. However, the sample included individuals from five provinces presenting various perspectives on the healthcare system, which helped provide a diversity in responses. We were only able to recruit a limited number of older adults with frailty and family caregivers, all of them from either Quebec or British Columbia. Our recruitment of older adults living with frailty using posters in geriatric clinics proved ineffective, possibly due to difficulty reading or understanding the poster, or poor health [55]. Their recruitment through health professionals, although more effective, has also not made it possible to recruit enough to ensure saturation within this population. This report might, therefore, not fully represent the perspectives of older adults with frailty and caregivers across the country. We did, however, meet with HCPs who had considerable experience in caring for older adults in each province.

Apart from patients, the majority of our participants were women. Therefore, a better representation of men in the sample might have created a different picture. In particular, given that women experience higher rates of frailty, our study misses the perspectives of men caregivers who play a crucial role in the care of older adults with frailty.

## Conclusions

Our study looked at how older adults with frailty, family caregivers, HCPs, health administrators, and decision makers prioritize clinical quality indicators in the context of frailty care. Taken as a whole, our results point to the need for a patient-centred care for older adults with frailty that is oriented toward quality of life. In addition to Patient-Reported Outcome Measures (PROMs) and Patient-Reported Experience Measures (PREMs), our results also highlight several indicators-such as healthcare staff skills, patients’ symptoms, family caregiver burden, and family doctor continuity of care-which have the potential to move care toward patient-centredness. Inclusion of these indicators into an audit and feedback mechanism could indeed drive improvements in the quality of care provided to older adults with frailty.

## Supplementary information

**Additional file 1: **Scoping review on the current healthcare services and models care offered to frail seniors 

**Additional file 2: **Expert panel consultation to select of quality indicators of the care provided to older adults with frailty, to be developed and extracted from administrative databases 

**Additional file 3: **Distribution of participant perceptions of the importance of the five clinical quality indicators for which significant differences between participants were measured (median; box: 25th, 75th percentiles, whiskers: 10th, 90th percentiles; dots: outliers)

## Data Availability

The datasets generated and/or analysed during the current study are available from the corresponding author on reasonable request.

## Abbreviations

QI: Quality indicator
HCPs: Healthcare providers
DMs: Healthcare administrators or policy/decision makers
