# Supplementary material for: Prioritization of indicators of the quality of care provided to older adults with frailty by key stakeholders from five canadian provinces
Source: BMC Geriatr. 2022 Feb 23;22:149. doi: 10.1186/s12877-022-02843-9 (PMC8864862; doi:10.1186/s12877-022-02843-9)
Supplement: Supplementary file 1 — Additional file 1: Scoping review on the current healthcare services and models care offered to frail seniors [file 12877_2022_2843_MOESM1_ESM.pdf]

Additional file 1.

Scoping review on the current healthcare services and models care offered to frail seniors

Objective

This scoping review aimed answering the following research questions: (1) what are the current healthcare services and models care offered to frail seniors? (2) What are the healthcare resource utilization and care outcomes for frail seniors? and (3) What factors influence the healthcare resource utilization and care outcomes of frail seniors? We used a recognized scoping review methodology to answer these questions,[1 2] and reviewed three types of reports about Canadian frail seniors: reports evaluating the impact of an intervention on the clinical quality of FS care (Type-I), reports describing an innovative intervention or model to improve FS care without any formal evaluation (Type-II), and reports describing the quality of care across various FS cohorts (Type-III). For each of the three report types, we identified and extracted indicators of the clinical quality of frail senior care.

Methods

Inclusion criteria

Types of reports

We included reports targeting Canadian FS of any type and format: empirical studies of any method (quantitative, qualitative or mixed methods) or design (descriptive, case study, before-and-after, randomized trials), national and provincial quality-related projects, programs, strategic plans, or initiatives that targeted FS. We also included reports on healthcare resource utilization and healthcare outcomes from administrative databases of FS in Canada. If a report covered both the Canadian population, and a population from another country, then it was included provided that the information (data or discussion) on Canadian FS could be extracted. We included reports published in French and/or English.

Types of participants

We included reports on FS aged 65+ years, their family caregivers, and/or their healthcare providers. For the purpose of the review, the participants studied in the reports were defined as being 'frail', (1) if they were described as frail, and the authors justified the use of the term 'frail' (e.g. by citing a frailty index), (2) if the description of participants provided in the report allowed us to class them as being frail using the *Canadian Study of Health and Aging* (CSHA) Clinical Frailty Scale,[3] **or** if two or more domains of the Edmonton Frail Scale[4] were described as health problems of the participants – meeting one scale or the other was required for inclusion, (3) if participants were described as living in long-term care facilities or palliative care home (hospices), or (4) if the participants were clearly identified to be at the end-of-life, terminally ill, or in palliative care. If a report covered both frail and not frail people, it was

included provided that the information on frail people could be extracted. Similarly, if a report covered both seniors and non-seniors, we included it if we could extract information (data or discussion) about frail seniors.

Types of clinical quality of care indicators

We only included reports that discussed at least one clinical quality indicator measure. Following the *Agency for Healthcare Research and Quality* taxonomy,[5] we searched for reports on the clinical quality of care of FS measured at levels of healthcare structure (macro), resources use and costs, process of healthcare services, and healthcare patient/caregiver/provider outcome. We also searched for qualitative information to identify potential reasons for variations in healthcare resource utilization and outcomes across patient cohorts, settings of care, or provinces and deepen our understanding of current healthcare services and models of care.

Search strategy

The population of the "frail seniors" can be described in many ways, with frailty having many signs and symptoms. The clinical characteristics of frailty are varied, and there are several different scales created to diagnose, describe or delineate frailty. At first, we tried identifying this patient population in the medical literature using clinical characteristics, but it resulted in a hugely sensitive search, encompassing most of the discipline of gerontology and nearly every description of interactions between the elderly and the medical system. Hence, to find articles describing models of care or best practices for the population as a whole, we chose to combine keywords indicative of frailty (frail*, vulnerab*, "at risk", "high risk", high-risk, "low function", dependent) near to keywords indicative of older age (older, elder*, senior*) in the titles or abstracts. This resulted in a highly targeted set of articles that discussed the population more directly. In addition to finding the articles which directly address the frail senior population, three further stipulations were used to select articles of interest. Firstly, currency was important, so the results were limited to articles published in the last 5 years. Next, any articles directly addressing the Canadian population where selected – any mention of Canada or any of its provinces or territories was used for this filter.

Electronic sources of data

We searched the following academic databases (2009- December 14th, 2014): Ageline, CINAHL, Cochrane Library, DARE, EMBASE, PsycINFO, Sociological Abstracts, PubMed, Ovid MEDLINE, and Ovid AMED. An experienced information scientist combined keywords from controlled vocabulary and free text to optimize the identification of relevant reports, and adapted this strategy for each database (Appendix 1). A second information scientist peer reviewed these search strategies. We also consulted with co-investigators, a geriatrician and a researcher specialized in the care of frail seniors to improve the specificity of the strategy.

For grey literature sources, we searched (January-March 2015) CUBIQ, AMICUS, SantéCom, CIHI, BAnQ, DIVA and the websites of the Canadian Hospice Palliative Care Association, open SIGLE, CADth, CORDIS, Evidence for Policy and Practice Information (EPPI) Centre, Database of promoting health effectiveness

reviews (DoPHER), Home and Community Care Digest, ICES, INESS, IUGM, NICE, OpenGrey (OpenSigle), PRISMA, IRSC-Fragilité et vieillissement, ProQuest Dissertations & Theses Full Text, UBC Centre for Health Services and Policy Research, UWaterloo-Geriatric Health Systems Research Group.

Other sources of data

We also searched the reference lists of primary articles and reviews, and ISI Web of Science and Google Scholar (in July 2015) for studies citing the most relevant reports included.

Selection of reports

The research team initially met to discuss decisions surrounding study inclusion and exclusion. Then, a calibration exercise with a random sample of approximately 100 reports ensured a proper training of reviewers, iteratively using 10 reports each time, until they reached a minimum level of 80% agreement. Two reviewers (among MAL, MBB, MM, NC, MAC, VVT) then read titles and abstracts of reports identified through the search strategy to assess which reports met the inclusion criteria. The team conducted a calibration exercise again before selecting reports based on the full text of those retained. After calibration, they were assessed by two reviewers to ensure they met the inclusion criteria. Disagreements regarding study inclusion were resolved by discussion between the two reviewers and when disagreements occurred, a third reviewer determined final inclusion.

Data extraction and management

For extraction, we used a web-based standardized data extraction form supported by specialized software (DistillerSR, Evidence Partners). We extracted data relative to the report (e.g. authors, date of publication), to the study (e.g. type of study, scope), and to the content (e.g. participant characteristics, description of intervention, impact, covariates, whether the report covers a 'hot topic' – defined as a theme of actuality). We categorized the included intervention (i.e. health, professional, financial, or regulatory interventions) according to the taxonomy developed by the Effective Practice and Organisation of Care editorial group of the Cochrane collaboration.[6] Extraction was initially iterative with continuous data extraction and updating of the data extraction form. Again, a calibration exercise with a random sample of approximately 20 reports ensured proper training of reviewers: two persons independently extracted data from the first five included studies and met to compare their results and determine whether their approach to data extraction was consistent with the research question and purpose. They extracted 5 reports at a time, iteratively, until they reached a minimum level of 80% agreement. Following this calibration, a first person completed the extraction of the remaining reports, a second person verified it, and when there was disagreement among them, a third senior reviewer (MM) made the final decision on the extraction or referred to the project leader (AMCG) to make a decision.

Analysis

Quantitative data

We prepared a descriptive numerical summary of the characteristics of the included reports (e.g.

number of studies included, type of author, language, province, targeted audience). We grouped and pooled data in tables according to the predefined Types of report (I- impact of intervention; II- description of intervention; III- comparison of various FS cohorts), type of setting, patient characteristics, province, and study design. We estimated frequency of each variable using SAS (version 9.4). For the Type-I reports evaluating the impact of an intervention or Type-II that described an intervention, we presented the clinical quality indicator (CQI) targeted by the intervention, a short description of the intervention, the categories of intervention and the impact of the intervention (statistically significant improvement, deterioration or no significant effect). For the Type-III reports comparing various FS cohorts, we presented the CQI targeted in the report, the covariates studied and their association to the CQI.

Qualitative data

Open-text fields of the extraction form were analyzed qualitatively using an inductive thematic analysis approach. We then articulated the findings, first through the three report types that correspond to the environmental scan aims, and then through sub-themes that emerged during extraction and coding. Lastly, the research team met to consider the meaning of the findings as they relate to the aims, and discuss implications for future research, practice, and policy.

Results

Search results

Using the academic databases we found 453 reports describing current healthcare services and outcomes to be considered for inclusion to the review (Figure 1.1). The grey literature search identified 580 further references, and 17 additional references were identified from the other sources. After removal of duplicates, 1004 references were retained for consideration for inclusion in the review.

Study selection

We included 93 studies: 22 studies of the impact of interventions to improve the quality of healthcare for FS, 35 describing an intervention (without a formal evaluation of their impact), and 42 comparing indicators of the quality of health care between various patient cohorts. Three of the studies comparing patient cohorts were also comprised among the intervention studies. Reasons for excluding reports comprised: not being written in French and/or English (N= 79), not covering the Canadian health care system (N = 599), not targeting seniors (N=604), not targeting frail seniors (n=850), and/or not being one of the three types of reports included (N= 117).

Description of the reports

Most of the included reports were published in 2012 (N=27), followed by 2013 (N=18) and 2014 (N=14). They were more frequently authored by researchers (N= 86) and/or clinicians (N= 66), and the authors were often based in universities (N= 82). Most reports were written in English (N= 85), whereas a small number were in French (N= 7); only one was written in both English and French. The conclusions of the included reports more often applied to the provincial level (N= 37), but some covered national (N= 11),

regional, and/or local levels (N= 45). Most of the included reports presented the results of an empirical study, which was defined here as new data collection or secondary analysis (review) (N= 86) (Table 1.1). Among the reports of an empirical study, most presented the results of quantitative descriptive studies without a control group (N=49/93), 7 presented randomized trials, 15 non-randomized studies with control group, 22 qualitative studies and 1 presented a literature review.

Ontario and Quebec were the provinces most often the focus of the work in the included reports (N=37 reports), followed by British Columbia, Nova Scotia, and Alberta (N=11 each). More reports covered settings of care outside of hospitals (N=70): most commonly in long-term care (N=26) and home care settings (N=21). Several reports also covered hospital settings (N=27), more often Geriatric care units and Emergency departments (N= 5). Most report targeted frail seniors (N=75/93), several targeted healthcare providers of diverse professions (N=25), and a few targeted caregivers and decision makers.

Frail seniors were most often recognized as being 'frail' through a classification by our research team using the description of participants provided in the report, by checking if they met the CSHA clinical frailty scale[3] or Edmonton Frail scale[7] (Table 1.2). This strategy allowed identifying 83% of the included reports. Only for 26/75 (35%) reports did the authors of the report cite a frailty index to justify that they classified participants as being 'frail'. Among the reports in which the participants were recognized as 'frail', based on a classification by the research team, several frailty domains were recognized by the team.

Among the studies that targeted FS, some reported the mean age of the FS included (N= 45/93 reports; mean = 81 years old, range = 72-87), others their median age (N=2/93 reports; median = 85; range = 85 – 86). Some reported the proportion of women (N =47/93 reports; mean = 67% women; range 0-100%). We also extracted if the report included mostly women (N= 40/93 reports), mostly men (N = 7/93), or if this information was not available. None of the included studies reported the proportion of FS belonging to a specific population group (e.g. Asian, Black, Filipino, Latin American, Aboriginal, or Caucasian). Only two reports described some of their frail senior participants as immigrants; the majority did not provide any information on the citizenship of the frail senior participants. Among the reports that targeted FS participants, most targeted FS living in a community (N=24/93 report), but several also targeted FS living alone (N=23), or living with a partner or caregiver (N=21). This information was lacking from many of the included reports (N=39) (Table 1.9). Most of the included reports did not specify if the targeted seniors were living in rural or urban settings (N=61/93 reports) – 10 targeted FS seniors living in rural settings, and 3 FS living in urban settings.

In the reports that targeted caregivers of FS, we did not find any information on the proportion actually living with FS.

The included reports covered several targeted 'hot topics' (Table 1.3).

Impact of interventions to improve FS care (Type-I)

Among the interventions that were formally evaluated in an experimental study (Type-I), there were health interventions (i.e. interventions targeting the patient's health) (N=14), organizational

interventions, (N=6), professional interventions (N=3), and one regulatory intervention (Table 1.4-A). Among those evaluating a health intervention, 11 reported a preventive intervention, five reported treatment interventions and one reported a screening intervention. Among the preventive interventions, exercise was the intervention most studied (N=4) and it generally improved the targeted clinical quality indicators, namely physical functioning, self-efficacy, cognitive performances, physical capacities (gait, balance, grip strength), and quality of life.[8-11] All the studied organizational interventions showed some benefits,[12-17] either on patient-level outcomes (e.g. increased empowerment or satisfaction with care, reduced unmet needs), healthcare provider outcomes (improved skills), resource utilization (e.g. increased number of home visits, reduced length of hospital stay, reduced ER use), or structure (institutional practices), although one also had negative impacts[13] on patient-level outcomes (unmet needs), caregiver (burden), resource use (ER and hospital use).

Innovative interventions described and not formally evaluated (Type-II)

The reports describing an innovative intervention or model to improve FS care without formally evaluating their impact (Type-II) (Table 1.4-B) included organizational interventions (N= 29), health interventions (N= 14), professional interventions (N=9) and financial interventions (N=2).

Quality of care across various frail senior cohorts (Type-III)

Analysis by clinical quality indicators (CQI)

The CQI most often compared across various frail senior cohort were: decreased mortality (N= 9), decrease in the rate of visits to the emergency department (N= 5), decrease in hospital use (N= 5), decrease in drug use (N= 4), and decrease in activities of daily living (N= 4) (Table 1.6).

Mortality was reported to decrease with increases in continuity of care (comprehensive of care, informational),[18] in males,[19] and in persons presenting with chronic obstructive lung disease.[20] On the other hand, increases in mortality were reported with frailty, [21-23] advanced disease (cancer),[23] comorbidities,[20] setting of care (LTC and ambulatory), history of falls,[20] use of psychotropic drugs[24] and symptoms and diseases (Parkinson, pressure ulcers, stroke).[20]

Rates of visits to the emergency department decreased with increasing age[25] and informational continuity of care.[18] However, rates of emergency visits increased in men,[26] in patients with cognitive impairment suspicion[27] and in those using hospital and LTC institutions.[28] No significant association was reported between the rate of visits to the ER and 11 covariates (age, sex, frailty, comorbidities, marital status, education, symptoms and diseases, advanced disease, extensive cancer treatment received continuity of care and neighborhood income).

Hospital use was significantly associated with 15 CQI. It decreased with local anesthesia (vs. general)[29] or in greater community size (ref: <10 000).[30] In contrast, a report showed that hospital use increased with increasing age, frailty, low strength social network, increasing number of medications, history of falls, health status (mild, moderate to high, ref: stable), and comorbidities.[30] Other reports also showed increases in hospital use in people who used long-term care,[31] with length of antibiotic prescriptions,[32] and with

increasing age.[33]

Men **used less drugs** than women.[33] However, covariates such as increasing age,[33] length of antibiotic prescription[32] and using long-term care settings[31] were associated with increases in drug use. No significant association was observed between drug use and neighborhood income, having a pharmacy chain affiliation (ref: no affiliation), LTC care or hospital use.

In a single report, the patients' **activities of daily living (ADL)** were demonstrated to increase with the patient's familiarity with home settings.[34] In contrast, other reports showed that ADL decreased or were unaffected by frailty[35-37] and by patient's familiarity with home settings.[34]

Analysis by covariate

In the reports describing the quality of care across various FS cohorts (Type-III), we identified 45 covariates associated with improvement, deterioration or without effect on CQIs. The covariates more frequently studied were frailty (N= 20), age (N= 16), sex (N= 11), comorbidities (N= 9) and setting of care (hospital and LTC) (N= 7 and N= 7; respectively). The covariates more frequently associated with improvement in CQI were frailty, age, sex, and setting of care (hospital use and LTC use).

Increasing level of frailty was associated with improvement in 5 CQI, deterioration in 14 CQI and no effect on 12 CQI. Higher frailty scores were associated with improvement in several CQI, notably decreased polypharmacy (patients needing constant assistance with eating and those who do not use toilet), increased quality-of-care processes in geriatric assessment (comprehensiveness, informational continuity, completion of advance health care directives), increased daily oral hygiene, increase in discharge process (ability to be discharged home for previously non-institutionalized patients), and increased decisions of the patient to decline scheduled treatment or procedure. Frailty was also reported to be associated with deteriorations in several CQI, notably, increased polypharmacy in patients sad or depressed, increased anxiety, increased depression, increased post-operative morbidity, increased mortality, increased risk of fracture and falls, increased cancer treatment toxicity, decreased physical function, decreased activities of daily living (ADL) and instrumental activities of daily living (IADL), increased hospital use, increased emergency department use and increased institutionalisation). Some reported a lack of association between frailty levels and mortality, cancer treatment toxicity, rate or number of fractures, risk of fall, activities of daily living (ADL), depression, polypharmacy, daily oral hygiene, number of medical visits, emergency department use, hospital use and institutionalization.

Increasing **age** was associated with improvement in four CQI (decrease in polypharmacy, decrease in the use of inpatient hospital services, decrease in emergency department use and increase in the quality-of-care process in geriatric assessment). Increasing age was also reported to be associated with deteriorations in four CQI (increase in institutionalisation, increase in hospital and drug use and decrease in the use of community based-care). No association was reported between age and 11 CQI (risk of fall, mortality, cancer treatment toxicity, depression, hospital admission or death within 30 days of treatment initiation, survival, activities of daily living (ADL), number of medical visits, emergency

department use, hospital and drug use).

Sex (being a woman) was associated with improvement in four CQI (decrease in polypharmacy, decrease in mortality, decrease in drug use and decrease in quality-of-care in geriatric assessment). In contrast, being a woman resulted in higher risk of institutionalization in LTC and use of emergency department while no significant association was observed between sex and seven CQI (mortality, cancer treatment toxicity, depression, survival, number of medical visits, emergency department use and hospital use).

The **number of comorbidities** was associated with a decrease in mortality. It was also associated with deteriorations in four CQI (increase in polypharmacy, increase in depression, increase in hospital admission or death within 30 days of treatment initiation, and decrease in survival)**.** Some of the included reports demonstrated a lack of association between age and six CQI (hospital admission or death within 30 days of treatment initiation, mortality, cancer treatment toxicity, emergency department use, hospital use and number of medical visits).

Setting of care (hospital or long-term care facilities) was associated with improvement in 5 CQI, deterioration in 7 CQI and was not associated with 5 CQI. Acute care hospital use was associated with increased survival, but it was also associated with deteriorations in four CQI (increased polypharmacy, increased institutionalization and increased hospital and emergency department use). No significant association was reported between hospital use and mortality. Hospital use was reported to be associated with increase, or decrease in quality of care processes in geriatric assessment, or to not be associated with it across various reports. Increases in the quality-of-care process in geriatric assessment were associated with hospital use defined as hospital length of stay, whereas decreased in the quality-of-care process in geriatric assessment were associated with hospital use defined as a previous admission to the Geriatric Assessment Unit (GAU), staying in a GAU for 2 weeks and discharge to community, home or independent-living facility. Long-term care (LTC) use was associated with improvement in a few CQI (decrease in polypharmacy and hospital admission or death within 30 days of treatment initiation), and in deterioration in three CQI (increase in drug use, increase in emergency department use and increase in mortality).

Clinical quality of care indicators

In this section, we describe the results regarding the six clinical quality of care indicators (CQI) that were prioritized by key stakeholders during the Activity 3 (interviews) of the present project: (1) increasing patient quality of life, (2) increasing providers' competencies or skills, (3) reducing symptoms, (4) reducing caregiver burden, (5) increasing patient satisfaction with care, and (6) increasing continuity of care by the family physician.

1 - Increasing patient quality of life

Overall, seven studies focused on patient quality of life. Four of them evaluated the impact of an intervention on quality of life (Table 1.5). Three interventions significantly increased patient quality of life (a 12-week small-group physical exercise program,[8] a multifactorial, interdisciplinary team

approach to falls prevention,[38] and a mobility intervention in long-term care facilities[39]). A single intervention had no significant impact on quality of life (assessment, collaborative care planning, health promotion, and referral to community health and social support services by homecare nurses).[40] Three additional studies described an intervention designed to improve quality of life, without formally evaluating its impact: one targeted increasing independence after illness,[41] one the improvement of oral health, [42] and one disease prevention.[43] None of the included studies described quality of life across various frail senior cohorts.

2- Increasing providers' competencies, skills, or knowledge

Overall, five reports presented interventions aiming to improve providers' competencies, skills, or knowledge. A single one, the VIDOS study, evaluated an intervention's impact on providers' competencies or skills (Table 1.12). It consisted of an interdisciplinary, multifaceted knowledge translation intervention, and it was associated with a significant improvement in providers' competency and skills. Four studies described interventions targeting provider competency and skills without reporting efficacy measures. Two proposed implementing an interprofessional model of care. The first involved a collaborative team that interacted during team-based case meetings, and aimed at enhancing interprofessional skills of geriatricians and primary care providers.[44] The second described an innovative interprofessional model offering secondary outpatients services for frail seniors (e.g. early diagnosis of dementia, assessment of complex medical conditions, and development of an interprofessional health improvement plan).[45] Team members attended training sessions and workshops organized by a regional clinical nurse. Two other interventions involved nurses working in hospitals or long-term care facilities. The first was a reflective practice intervention (RPI) including eight thematic workshops each 75 minutes long, delivered at three-week intervals, combined with reading assignments and individual exercises.[46] The RPI focused on three themes central to elder hospitalization: medication, mobilization, and discharge planning. The second was a comprehensive in-service education program for nurses and residential care-aide staff in provision of daily mouth care for elders in various long-term care facilities.[42] We found no studies describing this indicator across various frail senior cohorts.

3- Reducing symptoms

We identified 3 reports targeting a reduction of patients' symptoms. Two interventions did not have a significant effect on symptoms (a multifactorial, interdisciplinary team approach to falls prevention[38] and a nordic walking program[47]). A third report described an intervention consisting in a hierarchical assessment of balance and mobility, without studying its impact.[48] This intervention consisted of a bedside assessment of balance and mobility to help track acute changes in the health status of older people admitted to hospital. None of the included studies reported symptoms across various frail senior cohorts.

4- Reducing caregiver burden

Caregiver's burden was measured in a single study, the PRISMA study,[13] which evaluated the efficacy

of a coordination-type integrated service delivery model. The intervention was significantly associated with a deterioration of caregiver's burden. None of the included studies reported caregiver burden across various frail senior cohorts.

5- Increasing patient satisfaction with care

Four studies focused on patient satisfaction with care. Two of these evaluated the impact of an intervention. The PRISMA study showed an improvement in patient satisfaction with care after implementation of a coordination-type integrated service delivery model.[13] The other intervention evaluated the implementation of alternate housing models.[17] The descriptive studies consisted of an intervention regarding a dignity therapy for terminally ill patients [49] and the implementation of integrated models of care for frail seniors in Waterloo.[50] None of the included studies reported patient satisfaction with care in various frail senior cohorts.

6- Increasing continuity of care

None of the included reports discussed continuity of care as clinical quality indicators. It was only studied as a potential co-variate influencing other CQI.

References

1. Arksey H, O'Malley L. Scoping studies: towards a methodological framework. *International Journal of Social Research Methodology* 2005;8(1):19 - 32.
2. Levac D, Colquhoun H, O'Brien KK. Scoping studies: advancing the methodology. *Implement Sci* 2010;5:69. doi: 10.1186/1748-5908-5-69
3. Rockwood K, Song X, MacKnight C, et al. A global clinical measure of fitness and frailty in elderly people. *CMAJ* 2005;173(5):489-95. doi: 10.1503/cmaj.050051
4. Rolfson DB, Majumdar SR, Tsuyuki RT, et al. Validity and reliability of the Edmonton Frail Scale. *Age Ageing* 2006;35(5):526-9. doi: 10.1093/ageing/afl041
5. Agency for Healthcare Research and Quality. National Quality Measure Clearinghouse Rockville, USA: U.S. Department of Health & Human Services,; 2015 [updated June 18, 2015. Available from: http://www.qualitymeasures.ahrq.gov/index.aspx accessed March 2015 2014.
6. Effective Practice and Organisation of Care (EPOC). EPOC Taxonomy; 2002 [Available from: https://epoc.cochrane.org/epoc-taxonomy.
7. Ranking countries by quality of end of life care 2010 [Available from: http://www.economist.com/node/16585127?story_id=16585127&fsrc=rss accessed Sept. 3rd 2014.
8. Langlois F, Vu TT, Chasse K, et al. Benefits of physical exercise training on cognition and quality of life in frail older adults. *J Gerontol B Psychol Sci Soc Sci* 2012;68(3):400-4. doi: 10.1093/geronb/gbs069
9. Stolee P, Zaza C, Schuehlein S. Evaluation of a volunteer-led in-home exercise program for home-bound older adults. *Work* 2012;41(3):339-54. doi: 10.3233/WOR-2012-1304
10. Tousignant M, Corriveau H, Roy P-M, et al. Efficacy of supervised Tai Chi exercises versus conventional physical therapy exercises in fall prevention for frail older adults: a randomized controlled trial. *Disability and rehabilitation* 2013;35(17):1429-35.
11. Tousignant M, Corriveau H, Roy PM, et al. The effect of supervised Tai Chi intervention compared to a physiotherapy program on fall-related clinical outcomes: a randomized clinical trial. *Disabil Rehabil* 2012;34(3):196-201. doi: 10.3109/09638288.2011.591891
12. Bandurchin A, McNally M, Ferguson-Paré M. Bringing back the house call: how an emergency mobile nursing service is reducing avoidable emergency department visits for residents in long-term care homes. *Nursing leadership (Toronto, Ont)* 2011;24(1):59.
13. Hebert R, Raiche M, Dubois MF, et al. Impact of PRISMA, a coordination-type integrated service delivery system for frail older people in Quebec (Canada): A quasi-experimental study. *J Gerontol B Psychol Sci Soc Sci* 2010;65B(1):107-18. doi: 10.1093/geronb/gbp027
14. Kennedy CC, Thabane L, Ioannidis G, et al. Implementing a Knowledge Translation Intervention in Long-Term Care: Feasibility Results From the Vitamin D and Osteoporosis Study (ViDOS). *Journal of the American Medical Directors Association* 2014;15(12):943-45.
15. Prasad S, Dunn W, Hillier LM, et al. Rural geriatric glue: a nurse practitioner-led model of care for enhancing primary care for frail older adults within an ecosystem approach. *J Am Geriatr Soc* 2014;62(9):1772-80. doi: 10.1111/jgs.12982
16. Dubuc N, Dubois MF, Raiche M, et al. Meeting the home-care needs of disabled older persons living in the community: does integrated services delivery make a difference? *BMC Geriatr* 2011;11:67. doi: 10.1186/1471-2318-11-67 [published Online First: 2011/10/28]
17. DUBUC N, DUBOIS MF, DEMERS L, et al. Alternate housing models for older people with disabilities: their clientele, structures and resources in relation to the quality of and satisfaction with care delivery. *Ageing and Society* 2014;34(9):1575 - 600. doi: 0.1017/S0144686X13000354
18. Kergoat MJ, Latour J, Lebel P, et al. Quality-of-care processes in geriatric assessment units: principles,

practice, and outcomes. *J Am Med Dir Assoc* 2012;13(5):459-63. doi: 10.1016/j.jamda.2011.11.004
19. Heyland D, Cook D, Bagshaw SM, et al. The Very Elderly Admitted to ICU: A Quality Finish? *Crit Care Med* 2015;43(7):1352-60. doi: 10.1097/CCM.0000000000001024
20. Seitz DP, Anderson GM, Austin PC, et al. Effects of impairment in activities of daily living on predicting mortality following hip fracture surgery in studies using administrative healthcare databases. *BMC Geriatr* 2014;14:9. doi: 10.1186/1471-2318-14-9
21. Graham MM, Galbraith PD, O'Neill D, et al. Frailty and outcome in elderly patients with acute coronary syndrome. *Can J Cardiol* 2013;29(12):1610-5. doi: 10.1016/j.cjca.2013.08.016
22. Montero-Odasso M, Bergman H, Beland F, et al. Identifying mobility heterogeneity in very frail older adults. Are frail people all the same? *Arch Gerontol Geriatr* 2009;49(2):272-7. doi: 10.1016/j.archger.2008.09.010
23. Puts MT, Monette J, Girre V, et al. Are frailty markers useful for predicting treatment toxicity and mortality in older newly diagnosed cancer patients? Results from a prospective pilot study. *Crit Rev Oncol Hematol* 2011;78(2):138-49. doi: 10.1016/j.critrevonc.2010.04.003
24. Bronskill SE, Rochon PA, Gill SS, et al. The relationship between variations in antipsychotic prescribing across nursing homes and short-term mortality: quality of care implications. *Med Care* 2009;47(9):1000-8. doi: 10.1097/MLR.0b013e3181a3943f
25. Barbera L, Elit L, Krzyzanowska M, et al. End of life care for women with gynecologic cancers. *Gynecol Oncol* 2010;118(2):196-201. doi: 10.1016/j.ygyno.2010.04.014
26. Au A, Puts MT, Fletcher JD, et al. Frailty markers predicting emergency department visits in a community-dwelling sample of vulnerable seniors in Montreal. *Can J Aging* 2011;30(4):647-55. doi: 10.1017/S0714980811000511
27. Puts MT, Monette J, Girre V, et al. Does frailty predict hospitalization, emergency department visits, and visits to the general practitioner in older newly-diagnosed cancer patients? Results of a prospective pilot study. *Crit Rev Oncol Hematol* 2010;76(2):142-51. doi: 10.1016/j.critrevonc.2009.10.006
28. Gruneir A, Bronskill S, Bell C, et al. Recent health care transitions and emergency department use by chronic long term care residents: a population-based cohort study. *J Am Med Dir Assoc* 2012;13(3):202-6. doi: 10.1016/j.jamda.2011.10.001
29. Seitz DP, Gill SS, Bell CM, et al. Postoperative medical complications associated with anesthesia in older adults with dementia. *J Am Geriatr Soc* 2014;62(11):2102-9. doi: 10.1111/jgs.13106
30. Hogan DB, Amuah JE, Strain LA, et al. High rates of hospital admission among older residents in assisted living facilities: opportunities for intervention and impact on acute care. *Open Med* 2014;8(1):e33-45.
31. Daneman N, Gruneir A, Newman A, et al. Antibiotic use in long-term care facilities. *J Antimicrob Chemother* 2011;66(12):2856-63. doi: 10.1093/jac/dkr395
32. Daneman N, Gruneir A, Bronskill SE, et al. Prolonged antibiotic treatment in long-term care: role of the prescriber. *JAMA Intern Med* 2013;173(8):673-82. doi: 10.1001/jamainternmed.2013.3029
33. Kennedy CC, Ioannidis G, Thabane L, et al. Osteoporosis prescribing in long-term care: impact of a provincial knowledge translation strategy. *Can J Aging* 2015;34(2):137-48. doi: 10.1017/S0714980815000057
34. Provencher V, Demers L, Gagnon L, et al. Impact of familiar and unfamiliar settings on cooking task assessments in frail older adults with poor and preserved executive functions. *Int Psychogeriatr* 2012;24(5):775-83. doi: 10.1017/S104161021100216X
35. Nikolova R, Demers L, Beland F. Trajectories of cognitive decline and functional status in the frail older adults. *Arch Gerontol Geriatr* 2009;48(1):28-34. doi: 10.1016/j.archger.2007.09.007
36. Provencher V, Demers L, Gelinas I. Factors that may explain differences between home and clinic meal preparation task assessments in frail older adults. *Int J Rehabil Res* 2012;35(3):248-55. doi: 10.1097/MRR.0b013e3283544cb8

37. Provencher V, Demers L, Gelinas I, et al. Cooking task assessment in frail older adults: who performed better at home and in the clinic? *Scand J Occup Ther* 2013;20(5):374-83. doi: 10.3109/11038128.2012.743586
38. Markle-Reid M, Browne G, Gafni A, et al. The effects and costs of a multifactorial and interdisciplinary team approach to falls prevention for older home care clients 'at risk' for falling: a randomized controlled trial. *Can J Aging* 2010;29(1):139-61. doi: 10.1017/S0714980809990377
39. Slaughter SE, Wagg AS, Jones CA, et al. Mobility of Vulnerable Elders study: effect of the sit-to-stand activity on mobility, function, and quality of life. *J Am Med Dir Assoc* 2015;16(2):138-43. doi: 10.1016/j.jamda.2014.07.020
40. Ploeg J, Brazil K, Hutchison B, et al. Effect of preventive primary care outreach on health related quality of life among older adults at risk of functional decline: randomised controlled trial. *BMJ* 2010;340:c1480. doi: 10.1136/bmj.c1480
41. Enhanced activation and restorative care. Healthcare Management Forum; 2012. Elsevier.
42. Dharamsi S, Jivani K, Dean C, et al. Oral care for frail elders: knowledge, attitudes, and practices of long-term care staff. *Journal of dental education* 2009;73(5):581-88.
43. Markle-Reid M, Browne G, Gafni A. Nurse-led health promotion interventions improve quality of life in frail older home care clients: lessons learned from three randomized trials in Ontario, Canada. *Journal of evaluation in clinical practice* 2013;19(1):118-31.
44. Moore A, Patterson C, White J, et al. Interprofessional and integrated care of the elderly in a family health team. *Canadian family physician* 2012;58(8):e436-e41.
45. Kadowaki L, Chow H, Metcalfe S, et al. Innovative model of interprofessional geriatric consultation: specialized seniors clinics. *Healthcare quarterly (Toronto, Ont)* 2013;17(3):55-60.
46. Dubé V. Développement, mise à l'essai et évaluation d'une intervention de pratique réflexive avec des infirmières oeuvrant auprès de personnes âgées hospitalisées. Université de Montreal 2012.
47. Figueiredo S. Nordic Walking: A New Training for Frail Elderly2009.
48. Hubbard RE, Eeles EM, Rockwood MR, et al. Assessing balance and mobility to track illness and recovery in older inpatients. *Journal of general internal medicine* 2011;26(12):1471-78.
49. Chochinov HM, Cann B, Cullihall K, et al. Dignity therapy: a feasibility study of elders in long-term care. *Palliat Support Care* 2012;10(1):3-15. doi: 10.1017/S1478951511000538
50. Developing an integrated system of care for frail seniors. Healthcare Management Forum; 2013. SAGE Publications.
51. Magill MK, Senf J. A new method for measuring continuity of care in family practice residencies. *The Journal of family practice* 1987;24(2):165-8. [published Online First: 1987/02/01]
52. Rosenberg T. Acute Hospital Use, Nursing Home Placement, and Mortality in a Frail Community-Dwelling Cohort Managed with Primary Integrated Interdisciplinary Elder Care at Home. *Journal of the American Geriatrics Society* 2012;60(7):1340-46.
53. Laforest S, Nour K, Gignac MA, et al. The role of social reinforcement in the maintenance of short-term effects after a self-management intervention for frail housebound seniors with arthritis. *Can J Aging* 2012;31(2):195-207. doi: 10.1017/S0714980812000025
54. Pereira SR, Chiu W, Turner A, et al. How can we improve targeting of frail elderly patients to a geriatric day-hospital rehabilitation program? *BMC geriatrics* 2010;10(1):82.
55. Campitelli MA, Rosella LC, Stukel TA, et al. Influenza vaccination and all-cause mortality in community-dwelling elderly in Ontario, Canada, a cohort study. *Vaccine* 2010;29(2):240-6. doi: 10.1016/j.vaccine.2010.10.049
56. Juby AG. The challenges of interpreting efficacy of hip protector pads in fracture prevention in high-risk seniors. *Clin Rheumatol* 2009;28(6):723-7. doi: 10.1007/s10067-009-1115-1
57. Bravo G, Dubois MF, Demers L, et al. Does regulating private long-term care facilities lead to better care? A

study from Quebec, Canada. *Int J Qual Health Care* 2014;26(3):330-6. doi: 10.1093/intqhc/mzu032
58. Wyatt CC. A 5-year follow-up of older adults residing in long-term care facilities: utilisation of a comprehensive dental programme. *Gerodontology* 2009;26(4):282-90.
59. Seitz DP, Gill SS, Gruneir A, et al. Effects of cholinesterase inhibitors on postoperative outcomes of older adults with dementia undergoing hip fracture surgery. *Am J Geriatr Psychiatry* 2011;19(9):803-13. doi: 10.1097/JGP.0b013e3181ff67a1
60. Dasgupta M, Rolfson DB, Stolee P, et al. Frailty is associated with postoperative complications in older adults with medical problems. *Arch Gerontol Geriatr* 2009;48(1):78-83. doi: 10.1016/j.archger.2007.10.007
61. (CIHI) CIfhi. Caring for Seniors With Alzheimer's Disease and Other Forms of Dementia. Executive Summary. Analysis in brief, 2010:21.
62. Szczerbinska K, Hirdes JP, Zyczkowska J. Good news and bad news: depressive symptoms decline and undertreatment increases with age in home care and institutional settings. *Am J Geriatr Psychiatry* 2012;20(12):1045-56. doi: 10.1097/JGP.0b013e3182331702
63. Wu Z, Schimmele CM, Chappell NL. Aging and late-life depression. *J Aging Health* 2012;24(1):3-28. doi: 10.1177/0898264311422599
64. Sirois MJ, Emond M, Ouellet MC, et al. Cumulative incidence of functional decline after minor injuries in previously independent older Canadian individuals in the emergency department. *J Am Geriatr Soc* 2013;61(10):1661-8. doi: 10.1111/jgs.12482
65. Bourgault-Fagnou MD, Hadjistavropoulos HD. Understanding health anxiety among community dwelling seniors with varying degrees of frailty. *Aging Ment Health* 2009;13(2):226-37. doi: 10.1080/13607860802380664
66. Gill DP, Zou GY, Jones GR, et al. Comparison of regression models for the analysis of fall risk factors in older veterans. *Ann Epidemiol* 2009;19(8):523-30. doi: 10.1016/j.annepidem.2009.03.012
67. Kennedy CC, Ioannidis G, Rockwood K, et al. A Frailty Index predicts 10-year fracture risk in adults age 25 years and older: results from the Canadian Multicentre Osteoporosis Study (CaMos). *Osteoporos Int* 2014;25(12):2825-32. doi: 10.1007/s00198-014-2828-9
68. Stewart SL. Daily oral hygiene in residential care. *Canadian Journal of Dental Hygiene* 2013;47(1)
69. Rochon PA, Gruneir A, Gill SS, et al. Older men with dementia are at greater risk than women of serious events after initiating antipsychotic therapy. *J Am Geriatr Soc* 2013;61(1):55-61. doi: 10.1111/jgs.12061
70. Kergoat MJ, Latour J, Presse N, et al. Predictors of quality-of-care processes in geriatric assessment units: toward a better organizational framework. *J Am Med Dir Assoc* 2012;13(8):739-43. doi: 10.1016/j.jamda.2012.07.007
71. Moorhouse P, Mallery LH. Palliative and Therapeutic Harmonization: A Model for Appropriate Decision-Making in Frail Older Adults. *Journal of the American Geriatrics Society* 2012;60(12):2326-32.
72. Légaré F, Stacey D, Brière N, et al. Healthcare providers' intentions to engage in an interprofessional approach to shared decision-making in home care programs: a mixed methods study. *Journal of interprofessional care* 2013;27(3):214-22.

Figures and tables

Figure 1.1: Systematic review flow diagram

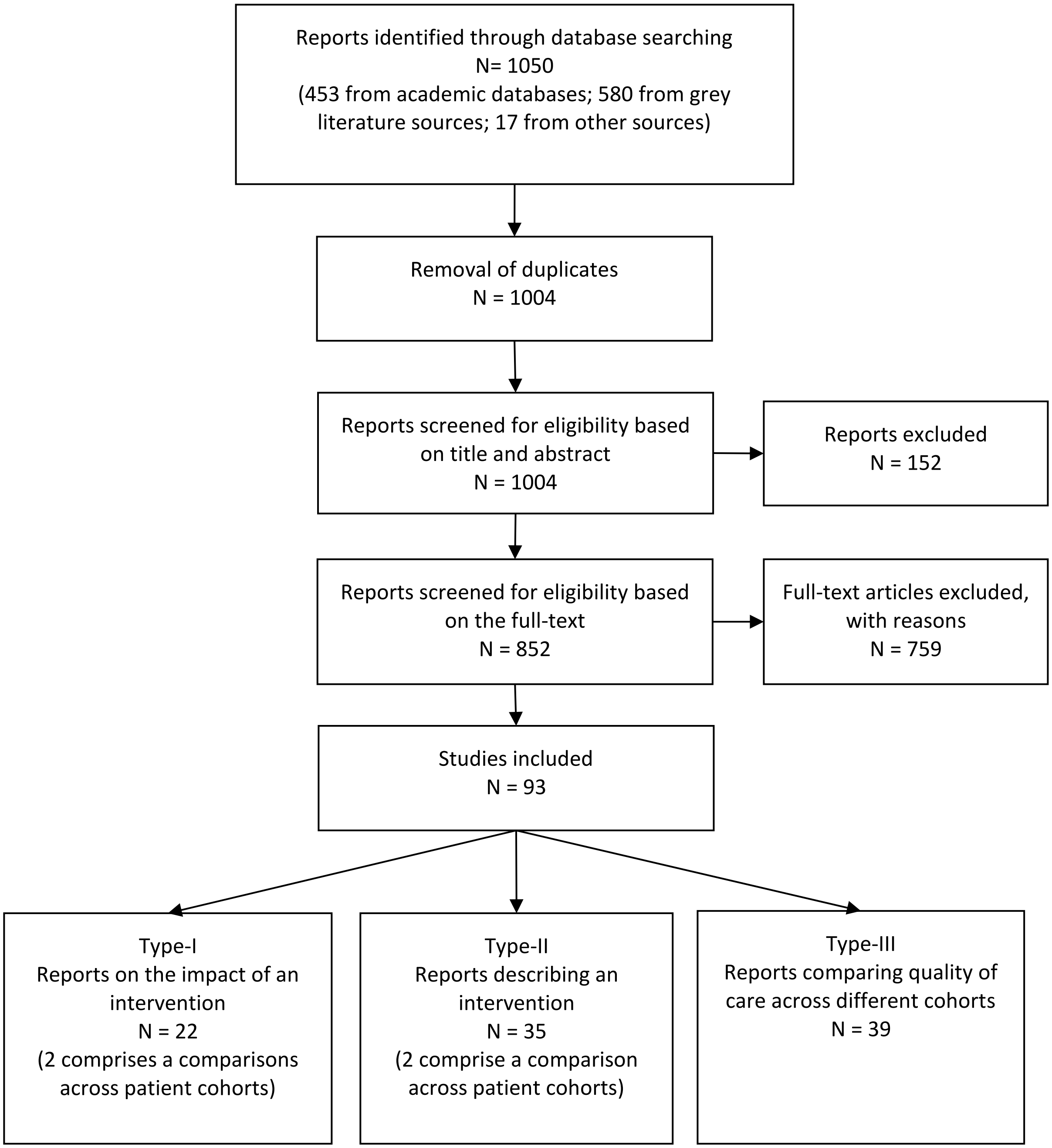

Table 1.1: Frequency of reports by study design (N= 93) (answers are not mutually exclusive).

Design	Frequency
Not an empirical study	7
Empirical studies	86
RCT (Randomized trial)	*7*
NRS (Non-randomized study with control group)	*15*
QDS (Quantitative descriptive study w/o control group)	*49*
QS (Qualitative study)	*22*
Review and/or meta-analysis (with quantitative data) (Specify type)	*1*
Review (qualitative data)(Specify type)	*0*

Table 1.2: Frequency of reports that mainly targeted FS by the criteria that the participants met to be identified as 'frail' (N=75). Reviewers were instructed to check all the items that apply (answers are not mutually exclusive).

Criterion met by participants described in report to be characterized as 'frail'	Frequency (%)
The description of participants provided in the report allows to classify them as frail using the *Canadian Study of Health and Aging* clinical frailty scale[51] or if two or more domains of the Edmonton Frail scale[4] were described as health problems of the targeted participant	62 (83%)
Participant are described as being 'frail' by the authors of the report, and the authors justify the use of the term 'frail' by citing an index.	26 (35%)
Participant are living in long term care facilities or palliative care home (hospices)	19 (25%)
Participant are clearly identified to be at the end-of-life, terminally ill, in palliative care	6 (8%)

Table 1.3: Frequency of reports covering targeted 'hot topics' (Total N= 93).

'Hot topic'	Frequency
Patient-centered care, patient/caregiver engagement, support, empowerment, shared decision making, communication	69
Care across the continuum, continuity of care (care transitions, appropriate care settings, information continuity across providers and settings, patient and family management through the system)	41
Health technology, innovative methods or strategies	28
Decision making (techniques)	25
Professional training, education, competencies	17
Health policy	15
Knowledge translation tools	14
Patient/Caregiver education and empowerment	8
Healthcare disparities	7
De-intensification of care, non-beneficial treatment (medical futility)	6
Advance care planning (directives)	5
Ethics	2
Inequities in health care	1
Place of death	1
Attitude toward death	1
Legislation / Jurisprudence	1
Enteral nutrition	1
Bereavement	0
Patient advocacy / Patient rights	0
Spirituality, value of life	0
Death certificate	0
Euthanasia	0
Volunteers	0

Table 1.4: Categories of interventions studied

A- Reports on the impact of an interventions (n=22) (type-I reports)

Intervention category		N
**Health intervention (n=14)**	Prevention	11
	Treatment	5
	Screening	1
	Other (Physical exercise training)	1
**Organisational intervention (n=6)**	Providers	6
**Professional intervention (n=3)**	Educational meetings	3
	Educational material	2
	Audit Feedback	1
	Consensus	1
	Outreach visits	1
	Patient mediated	1
**Regulatory intervention (n= 1)**	Regulation of LTC residences in Quebec	1

B- Reports describing innovative interventions or models without a formal evaluation of impacts (n=35) (type-II reports)

Intervention Category		N
**Organisational intervention (n=29)**	Providers	28
	Patients	2
	Structure	2
**Health intervention (n=14)**	Prevention	6
	Treatment	5
	Screening	7
	Other- shared decision making	1
**Professional intervention (n=9)**	Educational meetings	4
	Educational material	3
	Consensus	2
	Outreach visits	2
	Audit Feedback	1
	Opinion leader	1
**Financial intervention (n= 2)**	Provider	2
	Patient	1

Table 1.5: Type-I reports on the impact of an intervention on CQI (N= 22).

Clinical quality indicator	Interventions (Study ID)	Impact*
Increase in quality of life of the patient	12-week small-group physical exercise program [8]	☺
	Multifactorial, interdisciplinary team approach to falls prevention [38]	☺
	Mobility intervention in long-term care facilities [39]	☺
	Assessment, collaborative care planning, health promotion, and referral to community health and social support services by homecare nurses [40]	😐
Increase in health care staff skills	VIDOS study - Interdisciplinary, multifaceted knowledge translation intervention within long-term care (ON) [14]	☺
Decrease in symptoms	Multifactorial, interdisciplinary team approach to falls prevention [38]	😐
	Nordic walking (6 weeks of training, twice a week, 20 min daily) [47]	😐
Decrease in caregiver's burden (psychological, physical or financial costs experienced by a caregiver providing home care to a frail senior)	PRISMA study - Coordination-type integrated service delivery model (QC) [13]	☹
Increase in patient satisfaction with care	PRISMA study - Coordination-type integrated service delivery model (QC) [13]	☺
	Alternate housing models [17]	☺
Increase in family doctor continuity of care over the last year of life	None	
Decrease in risk of falling	Multifactorial, interdisciplinary team approach to falls prevention [38]	😐
Decrease in the rate of visits to the emergency department	Emergency mobile nursing service [12]	☺
	PRISMA study - Coordination-type integrated service delivery model (QC) [13]	☹
	Multifactorial, interdisciplinary team approach to falls prevention [38]	😐
	Primary integrated interdisciplinary elder care at home (PIECH) [52]	😐
Increase in the ability of patient to cope with difficulties, changes, and emotional struggles that arise with aging (coping effectiveness)	Social reinforcement for self-management of arthritis [53]	😐
Increase in patient empowerment (becoming self-sufficient)	PRISMA study - Coordination-type integrated service delivery model (QC) [13]	☺
Decrease in unmet needs of the patient	PRISMA study - Coordination-type integrated service delivery model (QC) [13]	☺
	PRISMA study - Coordination-type integrated service delivery model (QC) [16]	☺
	Alternate housing models [17]	😐
	12-week small-group physical exercise program [8]	☺

Clinical quality indicator	Interventions (Study ID)	Impact*
Increase in physical capacity (gait, balance)	The GDH rehabilitation program [54]	☺
	Nordic walking (6 weeks of training, twice a week, 20 min daily) [47]	☺
	Social reinforcement for self-management of arthritis [53]	☹
	Mobility intervention in long-term care facilities [39]	☹
	Multifactorial, interdisciplinary team approach to falls prevention [38]	😐
Decrease in hospital use	Primary integrated interdisciplinary elder care at home (PIECH) [52]	☺
	PRISMA study - Coordination-type integrated service delivery model (QC) [13]	☹
	Multifactorial, interdisciplinary team approach to falls prevention [38]	😐
Decrease in unmet needs of the patient	PRISMA study - Coordination-type integrated service delivery model (QC) [13]	☺
	PRISMA study - Coordination-type integrated service delivery model (QC) [16]	☺
	Alternate housing models [17]	😐
Increase in physical function	Mobility intervention in long-term care facilities [39]	😐
	Nordic walking (6 weeks of training, twice a week, 20 min daily) [47]	😐
	In-home volunteer-led exercise program [9]	☺
Increase in mental function (cognitive performance)	12-week small-group physical exercise program [8]	☺
	Multifactorial, interdisciplinary team approach to falls prevention [38]	😐
Increase in survival	Influenza vaccines [55]	☺
	Hip protector pads [56]	😐
Decrease in the rate of falls	Hip protector pads [56]	😐
	Multifactorial, interdisciplinary team approach to falls prevention [38]	😐
Decrease in the use of inpatient hospital services, such as receiving short-term treatment for a severe injury or episode of illness, an urgent medical condition, or during recovery from surgery	Primary integrated interdisciplinary elder care at home (PIECH) [52]	☺
	Multifactorial, interdisciplinary team approach to falls prevention [38]	😐
Increase quality of care	Regulation of private LTC residences in Quebec [57]	☺
	Alternate housing models [17]	☺
Improve oral health	Comprehensive dental program [58]	☺
Increase physical performance (grip strength)	PRISMA study - Coordination-type integrated service delivery model (QC) [13]	☺
Increase self-efficacy	Supervised Tai Chi [11]	☺
Increase in patient independence (autonomy)	Multifactorial, interdisciplinary team approach to falls prevention [38]	😐

Clinical quality indicator	Interventions (Study ID)	Impact*
Functional decline	PRISMA study - Coordination-type integrated service delivery model (QC) [13]	😐
Decrease risk of death	Assessment, collaborative care planning, health promotion, and referral to community health and social support services by homecare nurses [40]	😐
	PRISMA study - Coordination-type integrated service delivery model (QC) [13]	😐
Decrease in social isolation of the patient (social vulnerability)	12-week small-group physical exercise program [8]	☹
Decrease in depression (having the blues)	Multifactorial, interdisciplinary team approach to falls prevention [38]	😐
Increase in nutritional status	Multifactorial, interdisciplinary team approach to falls prevention [38]	😐
Decrease in number of medical visits	PRISMA study - Coordination-type integrated service delivery model (QC) [13]	☺
Decrease in the number of visits to specialists at a clinic during the last year of life	Care for senior model – Innovative model to improve care coordination and integration between specialists and acute care resources (ON)[15]	😐
Decrease in number of home visits	Care for senior model – Innovative model to improve care coordination and integration between specialists and acute care resources (ON)[15]	☺
Decrease in number of admissions to complex continuing care unit (CCC)	Care for senior model – Innovative model to improve care coordination and integration between specialists and acute care resources (ON)[15]	☺
Decrease in length of stay in complex continuing care unit (CCC)	Care for senior model – Innovative model to improve care coordination and integration between specialists and acute care resources (ON)[15]	😐
Decrease in the number of placements in long-term care/nursing homes	PRISMA study - Coordination-type integrated service delivery model (QC) [13]	😐
Improve institutional practices	Alternate housing models [17]	☺
Increase in prescription rates for osteoporosis prevention (vitamin D, calcium, osteoporosis medication)	Knowledge translation intervention (interactive educational meetings over 12 months) targeting fracture prevention in long term care [33]	☺
Decrease in patient helplessness (feeling of weakness)	Social reinforcement for self-management of arthritis [53]	😐

*: ☺ = significant improvement in clinical quality indicator (CQI), 😐 = no significant impact on CQI; ☹: significant deterioration in CQI

Table 1.6: Type-III reports describing variation in the quality of care across various FS cohorts (N= 39). Clinical quality of care indicators are ordered according to key stakeholders' ratings, from most to least important.

Clinical quality indicators (CQI)	Covariate	Impact*
Decreasing mortality	Frailty (Edmonton Frail Scale >=7)[21]	☹
	Continuity of care (comprehensive of care, informational)[18]	☺
	Continuity of care (all items of patient-centered care)[18]	😐
	Frailty (severe vs. moderate, mild mobility profile) [22]	☹
	Frailty (moderate vs. mild mobility profile)[22]	😐
	Frailty, sex (ref: female), comorbidities, symptoms and disease, extensive cancer treatment received [23]	😐
	Frailty (ADL disability; ECOG ≥ 2, ref: 0), advanced disease (cancer)[23]	☹
	Type of anesthesia (regional vs. general)[29]	😐
	Cholinesterase inhibitors users (vs. non-users)[59]	😐
	Symptoms and diseases (chronic obstructive lung disease)[20]	☺
	Comorbidities (number of major aggregated diagnosis group (ADGs: 3, ref: ≥ 5), setting of care (ambulatory, LTC), history of falls, symptoms and diseases (Parkinson, pressure ulcers, stroke)[20]	☹
	Age, sex (ref: male), comorbidities [(number of major aggregated diagnosis group (ADGs: 0, 1, 2, 4, ref: ≥ 5), Charlson comorbidity score (0, 1, 2, 3, ref: ≥ 4)], symptoms and diseases (congestive heart failure, ischemic heart disease, hearing impairment, osteoarthritis)[20]	😐
	Use of psychotropic drug[24]	☹
	Sex (ref: female)[19]	☺
	Age, comorbidities, health status, hospital use[19]	😐
Decrease in the rate of visits to the emergency department	Sex (ref: female)[26]	☹
	Age, frailty, comorbidities, marital status, education[26]	😐
	Age, sex (ref: male), frailty, comorbidities, symptoms and diseases, advanced disease (cancer), extensive cancer treatment received[27]	😐
	Cognitive impairment suspicion[27]	☹
	Continuity of care (comprehensiveness of care)[18]	😐
	Continuity (informational)[18]	☺
	Setting of care (hospital use, long-term care use)[28]	☹
	Increasing age[25]	☺

Clinical quality indicators (CQI)	Covariate	Impact*
	Neighborhood income[25]	😐
Decrease in hospital use	Frailty [22]	😐
	Age, sex (ref : female), frailty, comorbidities, symptoms and disease, advanced disease (cancer), extensive cancer treatment received [27]	😐
	Type of anesthesia (regional vs. General)[29]	☺
	Cholinesterase inhibitors users (vs. non-users) [59]	😐
	Greater community size (ref: <10 000)[30]	☺
	Age (≥ 90 yrs, ref: 65-79 yrs), frailty (fatigue moderate-severe vs. none, ADL score ≥ 3, ref : 0, walks with assistive device, uses wheelchair or scooter, ref: walks independently), family-social network (low strength of social relationships), hospital use (≥ 2 admissions in the past year), number of medications, history of falls, health status (mild, moderate to high, ref : stable) [30]	☹
	Age (80-90 yrs; ref: 65-79 yrs), frailty (fatigue: minimal vs. none, ADL score: 1,2 vs. 0), sex (ref : male), family-social network (few time involved in social activities, ref > 2/3 time), hospital use (1 admission in the past year), health status (mild to moderate, ref : stable), comorbidities [30]	😐
	Setting of care (long-term care use) [31]	☹
	Length of antibiotic prescription [32]	☹
	Age, neighbourhood income [25]	😐
	Sex (male) [33]	☺
	Age [33]	☹
	Decrease in drug use	😐
Decrease in drug use	Long-term care use[31]	☹
	Length of antibiotic prescription[32]	☹
	Age, neighborhood income[25]	😐
	Sex (male)[33]	☺
	Age[33]	☹
	Corporate chain affiliation (ref : no affiliation), long-term care use, profit status (municipal, non-profit, ref : for-profit)[33]	😐
Activities of daily living (ADL)	Frailty [35]	☹
	Environmental characteristics (familiarity with home settings – ADL process abilities measures)[34]	☺

Clinical quality indicators (CQI)	Covariate	Impact*
	Environmental characteristics (familiarity with home settings – ADL motor abilities measures)[34]	😐
	Frailty (balance problems, cognitive function assessed through the stroop-color naming test (independence), symptoms and disease (vision problems: glaucoma, cataract, macular degeneration or other disease-related vision loss)[36]	☹️
	Frailty (cognitive function assessed through stroop-interference tasks and stroop-color naming (adequacy), environmental characteristics, education[36]	😐
	Frailty (high score in the executive function test performance for ADL process ability measures, low grip strength), environmental characteristics (familiarity with home settings)[37]	☹️
	Frailty (high score in the executive function test performance for ADL motor ability measures)[37]	😐
Decrease post-operative morbidity	Frailty[60]	☹️
	Type of anesthesia (regional vs. general) [29]	😐
	Cholinesterase inhibitors users (vs. non-users) [59]	😐
Decrease in the number of placements in long-term care/nursing homes	Frailty (severe vs. moderate, mild mobility profile) [22]	☹️
	Frailty (moderate vs. mild mobility profile) [22]	😐
	Age, sex (ref : female), frailty, behaviours (physically abusive behavior, wandering, resistance to care), health status, marital status (unmarried), hospital use, use of psychotropic drug, symptoms and disease (no pain, diabetes) [61]	☹️
	Symptoms and disease (cancer, any incontinence) [61]	😐
Decrease in depression (having the blues)	Frailty (ADL score ≥ 2), health status (CHESS score ≥2), symptoms and disease (pain), comorbidities, number of medications [62]	☹️
	Age, symptoms and disease (any vision impairment), education, history of falls [62]	😐
	Age [63]	☺️
	Marital status (never married, ref : married), living in institution, visible minority, frailty (cognitive loss but no dementia, vascular dementia, other/unclassified dementia, ref: normal) [63]	☹️
	Sex (ref : female), frailty [ADL dependencies, dementia (probable Alzheimer's, possible Alzheimer's, ref: normal)], education, community size, marital status [63]	😐
Increase physical function (or executive function)	Frailty [35]	☹️
	Frailty [64]	☹️

Clinical quality indicators (CQI)	Covariate	Impact*
Increase in instrumental activities of daily living (IADL)	Frailty [35]	☹
Decrease in anxiety	Frailty, symptoms and disease [65]	☹
Decrease risk of recurrent community-acquired pneumonia	Cholinesterase inhibitors users vs. non-users [59]	😐
Decrease in the rate of frail seniors who experienced non-beneficial medical care during their last year of life (Resuscitation)	Cholinesterase inhibitors users vs. non-users [59]	😐
Decrease in risk of falling	History of fall, symptoms and disease (last vision examination), frailty (worsening of memory) [66]	☹
	Age, health status, symptoms and disease (foot problems), frailty (less physical activity) [66]	😐
Decrease in risk of fractures	Frailty [67]	☹
Decrease in the rate or number of fractures	Frailty [22]	😐
Increase in daily oral hygiene	Health status, frailty (ADL disabilities) [68]	☺
	Frailty, behaviors (aggressive), family-social network [68]	😐
Decrease polypharmacy	Age, sex (ref : female), use of long-term care (91-365 days vs. > 365 days), hospital use (no prior hospitalisation), facility level polypharmacy (low vs. expected level), behaviors (wandering, resistance to treatment), frailty (memory impairment, does not use the toilet, constant assistance with eating) [24]	☺
	Low income, weekly family contact, hospital use (acute care discharge within less than 30 days), prescribing physician, facility level polypharmacy (high vs. expected level), symptoms and disease (genitourinary disorders, neurological motor dysfunctioning, musculoskeletal disabilities, circulatory diseases, digestive disorders, pulmonary diseases), comorbidities, behaviors (anxious behavior, agitation), frailty (acts sad or depressed) [24]	☹
	Use of long-term care (≤ 90 days vs. > 365 days), symptoms and disease (infectious diseases, congenital anomalies, skin diseases, blood diseases, sensory disorders, neoplasms), behaviors (aggression), frailty (constant assistance with dressing, major assistance with transferring, assistance with toileting) [24]	😐
Decrease cancer treatment toxicity	Frailty (low grip strength), extensive cancer treatment received [23]	☹

Clinical quality indicators (CQI)	Covariate	Impact*
	Age, sex (female= 1), frailty, comorbidities, symptoms and disease (cancer), advanced disease (cancer) [23]	😐
Decrease in the rate of hospital admission or death within 30 days of treatment initiation	Setting of care (LTC vs. community) [69]	😊
	Comorbidities (≥ 2 vs. 0), use of psychotropic drug [69]	☹
	Age, comorbidities (1 vs. 0) [69]	😐
Decrease in the rate of hospital readmission	Continuity of care (informational) [70]	😊
	Continuity of care (comprehensiveness all items of patient-centered care) [70]	😐
Decrease in the number of medical visits	Age, frailty, sex (female = 1), comorbidities, symptoms and disease (cancer), advanced disease (cancer),, extensive cancer treatment received [27]	😐
Decrease in the use of acute inpatient hospital services, such as receiving short-term treatment for a severe injury or episode of illness, an urgent medical condition, or during recovery from surgery	Age [25]	😊
	Neighborhood income [25]	😐
Increase use of community-based care	Age [25]	☹
	Neighborhood income [25]	😐
Improve discharge processes (ability to be discharged home for previously non-institutionalized patients)	Frailty [60]	😊
Increase survival	Setting of care (hospital use) [19]	😊
	Comorbidities, health status [19]	☹
	Age, sex (ref : female) [19]	😐
Increase quality of care processes in geriatric assessment (comprehensiveness of care, informational continuity, completion of advance health care directives)	Age, sex (ref : male), history of falls, setting of care [(acute care only, ref: rehabilitation and acute care), university affiliation], profession of attending care provider (geriatrician), living situation before admission, frailty (cognitively impaired vs. not cognitively impaired), hospital use (patient hospital length of stay), high risk of mortality, contact with community health care professionals within 48 hours after admission [18]	😊
	Hospital use [(GAU target length of stay ≈2 weeks, ref: no target length of stay stated), GAU geographical location, previous admission to GAU in the past 6 months, patient discharged to community, home or independent-living facility], frailty (cognitive status not stated vs. not cognitively impaired) [18]	☹
	Hospital use (GAU target length of stay ≈ 3 weeks, ≈ 4 week, ref: no target length of stay stated), setting of care (rehabilitation care only, ref: rehabilitation and acute care) [18]	😐

Clinical quality indicators (CQI)	Covariate	Impact*
Improve decision of patient to decline scheduled treatment or procedure	Frailty [71]	☺
	Invasiveness of procedure [71]	😐
Increase in the intention to engage in an inter-professional approach to shared decision-making	Subjective norm (home support workers, nurses, social workers), perceived behavioural control (home support workers, nurses, social workers), cognitive attitude (home support workers), affective attitude (rehabilitation) [72]	☺
	Subjective norm (rehabilitation team), perceived behavioural control (rehabilitation team), cognitive attitude (nurses, social workers, rehabilitation), affective attitude (home support workers, nurses, social workers) [72]	😐
Increase in quality of life of the patient	None	
Increase in health care staff skills	None	
Decrease in symptoms	None	
Decrease in caregiver's burden (psychological, physical or financial costs experienced by a caregiver providing home care to a frail senior)	None	
Increasing in patient satisfaction with care	None	

*: ☺ = significant improvement in clinical quality indicator (CQI), 😐 = no significant impact on CQI; ☹: significant deterioration in CQI

Appendix 1: Academic Databases Search

A- Strategy for Ovid MEDLINE (search date: 2014/12/14)*

#	Query	Results
1	((frail* or vulnerab* or at risk or high risk or high-risk or low function or dependent) adj2 (older or elder* or senior*)).ti,ab.	8172
2	limit 1 to yr="2009 -Current"	3414
3	exp Nunavut/	196
4	exp Northwest Territories/	296
5	exp Yukon Territory/	142
6	exp British Columbia/	8071
7	exp Alberta/	6048
8	exp Saskatchewan/	2040
9	exp Manitoba/	2649
10	exp Ontario/	20944
11	exp Quebec/	11080
12	exp New Brunswick/	605
13	exp Nova Scotia/	2015
14	exp Prince Edward Island/	252
15	exp Newfoundland/	1107
16	exp Canada/	124714
17	or/3-16	124714
18	meta-analysis.pt.	55011
19	meta-analysis/ or systematic review/ or meta-analysis as topic/ or "meta analysis (topic)"/ or "systematic review (topic)"/ or exp technology assessment, biomedical/	78018
20	((systematic* adj3 (review* or overview*)) or (methodologic* adj3 (review* or overview*))).ti,ab.	71952
21	((quantitative adj3 (review* or overview* or synthes*)) or (research adj3 (integrati* or overview*))).ti,ab.	6112
22	((integrative adj3 (review* or overview*)) or (collaborative adj3 (review* or overview*)) or (pool* adj3 analy*)).ti,ab.	12690
23	(data synthes* or data extraction* or data abstraction*).ti,ab.	15198
24	(handsearch* or hand search*).ti,ab.	6177
25	(mantel haenszel or peto or der simonian or dersimonian or fixed effect* or latin square*).ti,ab.	15273
26	(met analy* or metanaly* or technology assessment* or HTA or HTAs or technology overview* or technology appraisal*).ti,ab.	5400
27	(meta regression* or metaregression*).ti,ab.	3209
28	(meta-analy* or metaanaly* or systematic review* or biomedical technology assessment* or bio-medical technology assessment*).mp,hw.	135212
29	(medline or cochrane or pubmed or medlars or embase or cinahl).ti,ab,hw.	103098
30	(cochrane or (health adj2 technology assessment) or evidence report).jw.	15678
31	(comparative adj3 (efficacy or effectiveness)).ti,ab.	7463
32	(meta-analysis or systematic review).mp.	123877
33	(outcomes research or relative effectiveness).ti,ab.	6090
34	((indirect or indirect treatment or mixed-treatment) adj comparison*).ti,ab.	1085
35	or/18-34	241194
36	2 and 17 [Canada]	87
37	2 and 35 [Reviews]	201

B- Strategy for Ovid AMED (Allied and Complementary Medicine) (search date: 2014/12/14) 1985 to December 2014

#	Query	Results
1	((frail* or vulnerab* or at risk or high risk or high-risk or low function or dependent) adj2 (older or elder* or senior*)).ti,ab.	713
2	limit 1 to yr="2009 -Current"	169
3	exp Nunavut/	0
4	exp Northwest Territories/	0
5	exp Yukon Territory/	0
6	exp British Columbia/	0
7	exp Alberta/	0
8	exp Saskatchewan/	0
9	exp Manitoba/	0
10	exp Ontario/	0
11	exp Quebec/	0
12	exp New Brunswick/	0
13	exp Nova Scotia/	0
14	exp Prince Edward Island/	0
15	exp Newfoundland/	0
16	exp Canada/	1256
17	or/3-16	1256
18	meta-analysis.pt.	333
19	meta-analysis/ or systematic review/ or meta-analysis as topic/ or "meta analysis (topic)"/ or "systematic review (topic)"/ or exp technology assessment, biomedical/	132
20	((systematic* adj3 (review* or overview*)) or (methodologic* adj3 (review* or overview*))).ti,ab.	2784
21	((quantitative adj3 (review* or overview* or synthes*)) or (research adj3 (integrati* or overview*))).ti,ab.	355
22	((integrative adj3 (review* or overview*)) or (collaborative adj3 (review* or overview*)) or (pool* adj3 analy*)).ti,ab.	186
23	(data synthes* or data extraction* or data abstraction*).ti,ab.	470
24	(handsearch* or hand search*).ti,ab.	209
25	(mantel haenszel or peto or der simonian or dersimonian or fixed effect* or latin square*).ti,ab.	81
26	(met analy* or metanaly* or technology assessment* or HTA or HTAs or technology overview* or technology appraisal*).ti,ab.	82
27	(meta regression* or metaregression*).ti,ab.	36
28	(meta-analy* or metaanaly* or systematic review* or biomedical technology assessment* or bio-medical technology assessment*).mp,hw.	3053
29	(medline or cochrane or pubmed or medlars or embase or cinahl).ti,ab,hw.	2030
30	(cochrane or (health adj2 technology assessment) or evidence report).jw.	0
31	(comparative adj3 (efficacy or effectiveness)).ti,ab.	98
32	(meta-analysis or systematic review).mp.	2629
33	(outcomes research or relative effectiveness).ti,ab.	231
34	((indirect or indirect treatment or mixed-treatment) adj comparison*).ti,ab.	4
35	or/18-34	5219
36	2 and 17 [Canada]	1
37	2 and 35 [Reviews]	8

C- Strategy for Embase (search date: 2014/12/14)

#	Query	Results
15	#2 AND #14	195
14	#5 OR #6 OR #7 OR #8 OR #9 OR #10 OR #11 OR #12 OR #13	244038
13	comparative NEAR/3 (efficacy OR effectiveness)	20226
12	meta NEXT/1 regression* OR metaregression*	3727
11	met NEXT/1 analy* OR metanaly* OR technology NEXT/1 assessment* OR hta OR htas OR technology NEXT/1 overview* OR technology NEXT/1 appraisal* 25,688#10 'mantel haenszel' OR peto OR 'der simonian' OR dersimonian OR fixed NEXT/1 effect* OR latin NEXT/1 square*	19187
9	handsearch* OR (hand AND search*)	13297
8	data NEXT/1 synthes* OR data NEXT/1 extraction* OR data NEXT/1 abstraction*	20328
7	integrative NEAR/3 (review* OR overview*) OR collaborative NEAR/3 (review* OR overview*) OR pool* NEAR/3 analy*	16571
6	quantitative NEAR/3 (review* OR overview* OR synthes*) OR research NEAR/3 (integrati* OR overview*)	21242
5	'systematic review'/exp OR 'meta-analysis'/exp	136605
4	#2 AND #3	75
3	'canada'/exp	131560
2	#1 AND [2009-2015]/py	5016
1	((frail* OR vulnerab* OR 'at risk' OR 'high risk' OR 'high-risk' OR 'low function' OR dependent) NEAR/2 (older OR elder* OR senior*)):ab,ti	10226

D- Strategy for Pubmed (search date: 2014/12/14)

#	Query	Results
7	Search (#3 and #6)	170
6	Search (systematic[sb] OR meta-analysis[pt] OR meta-analysis as topic[mh] OR meta-analysis[mh] OR meta analy*[tw] OR metanaly*[tw] OR metaanaly*[tw] OR met analy*[tw] OR integrative research[tiab] OR integrative review*[tiab] OR integrative overview*[tiab] OR research integration*[tiab] OR research overview*[tiab] OR collaborative review*[tiab] OR collaborative overview*[tiab] OR systematic review*[tiab] OR technology assessment*[tiab] OR technology overview*[tiab] OR "Technology Assessment, Biomedical"[mh] OR HTA[tiab] OR HTAs[tiab] OR comparative efficacy[tiab] OR comparative effectiveness[tiab] OR outcomes research[tiab] OR indirect comparison*[tiab] OR ((indirect treatment[tiab] OR mixed-treatment[tiab]) AND comparison*[tiab]) OR Embase*[tiab] OR Cinahl*[tiab] OR systematic overview*[tiab] OR methodological overview*[tiab] OR methodologic overview*[tiab] OR methodological review*[tiab] OR methodologic review*[tiab] OR quantitative review*[tiab] OR quantitative overview*[tiab] OR quantitative synthes*[tiab] OR pooled analy*[tiab] OR Cochrane[tiab] OR Medline[tiab] OR Pubmed[tiab] OR Medlars[tiab] OR handsearch*[tiab] OR hand search*[tiab] OR meta-regression*[tiab] OR metaregression*[tiab] OR data synthes*[tiab] OR data extraction[tiab] OR data abstraction*[tiab] OR mantel haenszel[tiab] OR peto[tiab] OR der-simonian[tiab] OR dersimonian[tiab] OR fixed effect*[tiab] OR "Cochrane Database Syst Rev"[Journal:__jrid21711] OR "health technology assessment winchester, england"[Journal] OR "Evid Rep Technol Assess (Full Rep)"[Journal] OR "Evid Rep Technol Assess (Summ)"[Journal] OR "Int J Technol Assess Health Care"[Journal] OR "GMS Health Technol Assess"[Journal] OR "Health Technol Assess (Rockv)"[Journal] OR "Health Technol Assess Rep"[Journal])	317335
5	Search (#3 and #4)	272
4	Search (canada or canadian*)	575166
3	Search (#1 and #2)	3806
2	Search (older[Title] OR elder*[Title] OR senior*[Title])	146625
1	Search (frail*[Title]) OR vulnerab*[Title]) OR at risk[Title]) OR high risk[Title]) OR high-risk[Title]) OR low function[Title]) OR dependent[Title])	223152

E- Search strategies for CINHAL (from 20141214)

((AB (frail* OR vulnerab* OR 'at risk' OR 'high risk' OR 'high-risk' OR 'low function' OR dependent) n2 (older OR elder* OR senior*)) OR (TI (frail* OR vulnerab* OR 'at risk' OR 'high risk' OR 'high-risk' OR 'low function' OR dependent) n2 (older OR elder* OR senior*))) AND (canada or canadian*)
Results: 80
(((AB (frail* OR vulnerab* OR 'at risk' OR 'high risk' OR 'high-risk' OR 'low function' OR dependent) n2 (older OR elder* OR senior*)) OR (TI (frail* OR vulnerab* OR 'at risk' OR 'high risk' OR 'high-risk' OR 'low function' OR dependent) n2 (older OR elder* OR senior*)))) AND review
Results: 375

F- Search strategy for DARE (search date: 2014/12/14)

((frail* or vulnerab* or 'at risk' or 'high risk' or 'high-risk' or 'low function' or dependent) near/2 (older or elder* or senior*))

Results: 46

G- Search strategies for PsycINFO (search date: 2014/12/14)

50 results for (Any Field:(review)) *AND* (Title:(((frail* OR vulnerab* OR 'at risk' OR 'high risk' OR 'highrisk' OR 'low function' OR dependent) NEAR/2 (older OR elder* OR senior*))) OR Abstract:(((frail* OR vulnerab* OR 'at risk' OR 'high risk' OR 'highrisk' OR 'low function' OR dependent) NEAR/2 (older OR elder* OR senior*))))

Results: 50

45 results for (Any Field:(canada or canadian*)) *AND* (Title:(((frail* OR vulnerab* OR 'at risk' OR 'high risk' OR 'highrisk' OR 'low function' OR dependent) NEAR/2 (older OR elder* OR senior*))) OR Abstract:(((frail* OR vulnerab* OR 'at risk' OR 'high risk' OR 'highrisk' OR 'low function' OR dependent) NEAR/2 (older OR elder* OR senior*))))

Results: 45

H- Search strategies for Sociological Abstracts (search date: 2014/12/14)

(ti((frail* OR vulnerab* OR 'at risk' OR 'high risk' OR 'high-risk' OR 'low function' OR dependent) AND (older OR elder* OR senior*)) OR ab((frail* OR vulnerab* OR 'at risk' OR 'high risk' OR 'high-risk' OR 'low function' OR dependent) AND (older OR elder* OR senior*))) AND (all(canada) OR all(canadian*))

Results: 124

(ti((frail* OR vulnerab* OR 'at risk' OR 'high risk' OR 'high-risk' OR 'low function' OR dependent) AND (older OR elder* OR senior*)) OR ab((frail* OR vulnerab* OR 'at risk' OR 'high risk' OR 'high-risk' OR 'low function' OR dependent) AND (older OR elder* OR senior*))) AND (all(review*))

Results: 513

I- Search strategies for Social Services Abstracts (search date: 2014/12/14)

(ti((frail* OR vulnerab* OR 'at risk' OR 'high risk' OR 'high-risk' OR 'low function' OR dependent) AND (older OR elder* OR senior*)) OR ab((frail* OR vulnerab* OR 'at risk' OR 'high risk' OR 'high-risk' OR 'low function' OR dependent) AND (older OR elder* OR senior*))) AND (all(canada) OR all(canadian*))

Results: 197

(ti((frail* OR vulnerab* OR 'at risk' OR 'high risk' OR 'high-risk' OR 'low function' OR dependent) AND (older OR elder* OR senior*)) OR ab((frail* OR vulnerab* OR 'at risk' OR 'high risk' OR 'high-risk' OR 'low function' OR dependent) AND (older OR elder* OR senior*))) AND (all(review*))

Results: 337

J- Search strategy for the Cochrane Library (search date: 2014/12/14)

((frail* or vulnerab* or 'at risk' or 'high risk' or 'high-risk' or 'low function' or dependent) near/2 (older or elder* or senior*)) 12

K- Search strategies for Ageline (search date: 2014/12/14)

((AB (frail* OR vulnerab* OR 'at risk' OR 'high risk' OR 'high-risk' OR 'low function' OR dependent) n2 (older OR elder* OR senior*)) OR (TI (frail* OR vulnerab* OR 'at risk' OR 'high risk' OR 'high-risk' OR 'low function' OR dependent) n2 (older OR elder* OR senior*))) AND (canada or canadian*)

Published Date: 20090101-20141231

Results: 37

(((AB (frail* OR vulnerab* OR 'at risk' OR 'high risk' OR 'high-risk' OR 'low function' OR dependent) n2 (older OR elder* OR senior*)) OR (TI (frail* OR vulnerab* OR 'at risk' OR 'high risk' OR 'high-risk' OR 'low function' OR dependent) n2 (older OR elder* OR senior*)))) AND review

Published Date: 20090101-20141231

Results: 107
