# Supplementary material for: Prioritization of indicators of the quality of care provided to older adults with frailty by key stakeholders from five canadian provinces
Source: BMC Geriatr. 2022 Feb 23;22:149. doi: 10.1186/s12877-022-02843-9 (PMC8864862; doi:10.1186/s12877-022-02843-9)
Supplement: Supplementary file 2 — Additional file 2: Expert panel consultation to select of quality indicators of the care provided to older adults with frailty, to be developed and extracted from administrative databases [file 12877_2022_2843_MOESM2_ESM.pdf]

Additional file 2.

Expert panel consultation to select of quality indicators of the care provided to older adults with frailty, to be developed and extracted from administrative databases

We sought consensus about which of the clinical quality indicators selected would be the most important/relevant to be developed and extracted from administrative databases, using a modified electronic Delphi survey

Preliminary list

We listed indicators of clinical quality relative to end of life (EoL) care that had been previously studied in Nova Scotia (NS) using administrative health databases.[1 2] To this initial list, we added the indicators extracted from the scoping review, and then selected the indicators that could be developed using common administrative healthdata. This preliminary list of indicators formed the basis of the first round of the Delphi process described below (Table 1: 'Round 1').

Delphi process to select priority indicators of clinical quality

Methods

We sought consensus about which of the clinical quality indicators selected would be the most important/relevant to be developed and extracted from administrative databases, using a modified electronic Delphi survey.[3 4] We invited the members of the project team, including members of the our stakeholder advisory group and research staff, to complete the survey, asking them to rank the preliminary list of indicators. In a first round, we asked the participants to rank the selected indicators based on their perceptions of their importance/relevance in assessing the performance of individual clinicians, clinical delivery teams or delivery organizations in the provision of care to older adults with frailty. Forced ranking ensured that each indicator was assigned a unique rank. During this first round, participants also had the opportunity to add other indicators they perceived as important for consideration. Following the first round of the Delphi survey, the median, minimum, and maximum ranks obtained for each indicator was computed. A teleconference meeting took place for team members to discuss the results and clarify any misunderstandings. In the subsequent rounds, we invited the participants to rank each indicator again according to their perceptions of its priority/relevance, but also considering the rank that each indicator obtained during the first round. Only those indicators that achieved a median rank of eight and above (i.e., 1-8) were included in the second round. We planned to stop the Delphi when stability was achieved in the ranks (less than a 15% change in voter distribution between one round to the next round of the Delphi survey).[5]

Results

Of 23 team members invited, 19 completed the Delphi questionnaire at first round and 21 completed the second round. Participants comprised two family physicians, 10 researchers, five decision makers,

two specialist physicians, two geriatricians and two research assistants (categories are not mutually exclusive). The participants required two rounds to reach consensus (Table 2.1). During the team meeting between the 2 rounds, it was decided that "Number of falls during last year of life" would not be included in the second round as this measure is not an indicator of clinical quality of care, but is instead a frailty indicator. A single indicator changed rank between the first and the second round: "Rate of Emergency Department visits" went from the 3rd to the 2nd rank. Stability of ranks was thus achieved after two ranks and the Delphi was stopped. The top ranking indicators (i.e., ranked 1, 2, or 3) were considered for indicator development.

The decision was made that when calculating the proportion of older adults with frailty that have undergone non-beneficial medical interventions during their last month of life, only ventilation would be considered. The decision to limit the indicator in this way was based on the difficulty of reliably identifying non-beneficial procedures within administrative data, which would represent a new study in itself.

In addition to the quality indicators selected through the Delphi, we also decided to extract several measures of health services utilization to help understand care delivery to older adults with frailty, including:

- New admissions to hospital
- Use of provider services (number of visits)
- Use of provider services by specialty (number of visits by specialty)

References

1. Experience of care during the end of life: A population-based Mortality Follow Back Study 2011 [Available from: http://www.dal.ca/content/dam/dalhousie/pdf/sites/nels/NELSnews_05.pdf.
2. Burge F, Lawson B, Johnston G, et al. Bereaved family member perceptions of patient-focused family-centred care during the last 30 days of life using a mortality follow-back survey: does location matter? *BMC Palliat Care* 2014;13:25. doi: 10.1186/1472-684X-13-25
3. Boulkedid R, Abdoul H, Loustau M, et al. Using and reporting the Delphi method for selecting healthcare quality indicators: a systematic review. *Plos One* 2011;6(6):e20476. doi: 10.1371/journal.pone.0020476 [published Online First: 2011/06/23]
4. Dalkey N, Helmer O. An experimental application of the Delphi method to the use of experts. *Management Science* 1963;9(3):458-67.
5. Scheibe M, Skutsh M, Schofer J. Experiments in Delphi Methodology. In: Linstone H, Turoff M, eds. The Delphi Method: Techniques and Applications2002:608.

Figures and tables

Table 1: Potential clinical quality of care indicators presented to our Delphi panel and median rank (legend: * = selected for extraction)

Clinical quality of care indicators	Median rank	
	Round 1	Round 2
– Number of hospital days during last year of life	4	1*
– Family physician continuity of care over the last year of life (note: index includes ambulatory visits only)	4	3*
– Number of ICU admissions during last year of life	5	3*
– Rate of Emergency Department visits (sum of ED visits over last year divided by the number of days out-of-hospital)	5	2*
– Proportion of frail seniors who have undergone non-beneficial medical interventions during their last month of life (a small number of non-beneficial treatments will be identified)	6	3*
– Number of new hospital admissions during last year of life	6	7
– Location where the frail senior spent the majority of their time during last year of life (proportion in each location, e.g. home, hospital, if data available LTC)	6	8
– Proportion of family physician visits over all physician visits during the last year of life	8	9
– Number of falls during last year of life	8	removed
– Proportion of frail seniors who received at least one physician house call during last year of life	9	removed
– Total number of family physician visits during last year of life	9	removed
– Total number of specialty ambulatory visits during last year of life	10	removed
– Proportion of frail seniors who are registered with a palliative care program	11	removed
**Additional indicators proposed by the panelists at first round**		
– Multidisciplinary care: proportion of ambulatory GP visits over all ambulatory visits during the last year of life	-	9
– Rate of inpatient readmission (readmission 1 week following last hospital separation)	-	3
