# Supplementary material for: Prioritization of indicators of the quality of care provided to older adults with frailty by key stakeholders from five canadian provinces
Source: BMC Geriatr. 2022 Feb 23;22:149. doi: 10.1186/s12877-022-02843-9 (PMC8864862; doi:10.1186/s12877-022-02843-9)

Additional file 3.

Distribution of participant perceptions of the importance of the five clinical quality indicators for which significant differences between participants were measured (median; box: 25th, 75th percentiles, whiskers: 10th, 90th percentiles; dots: outliers).

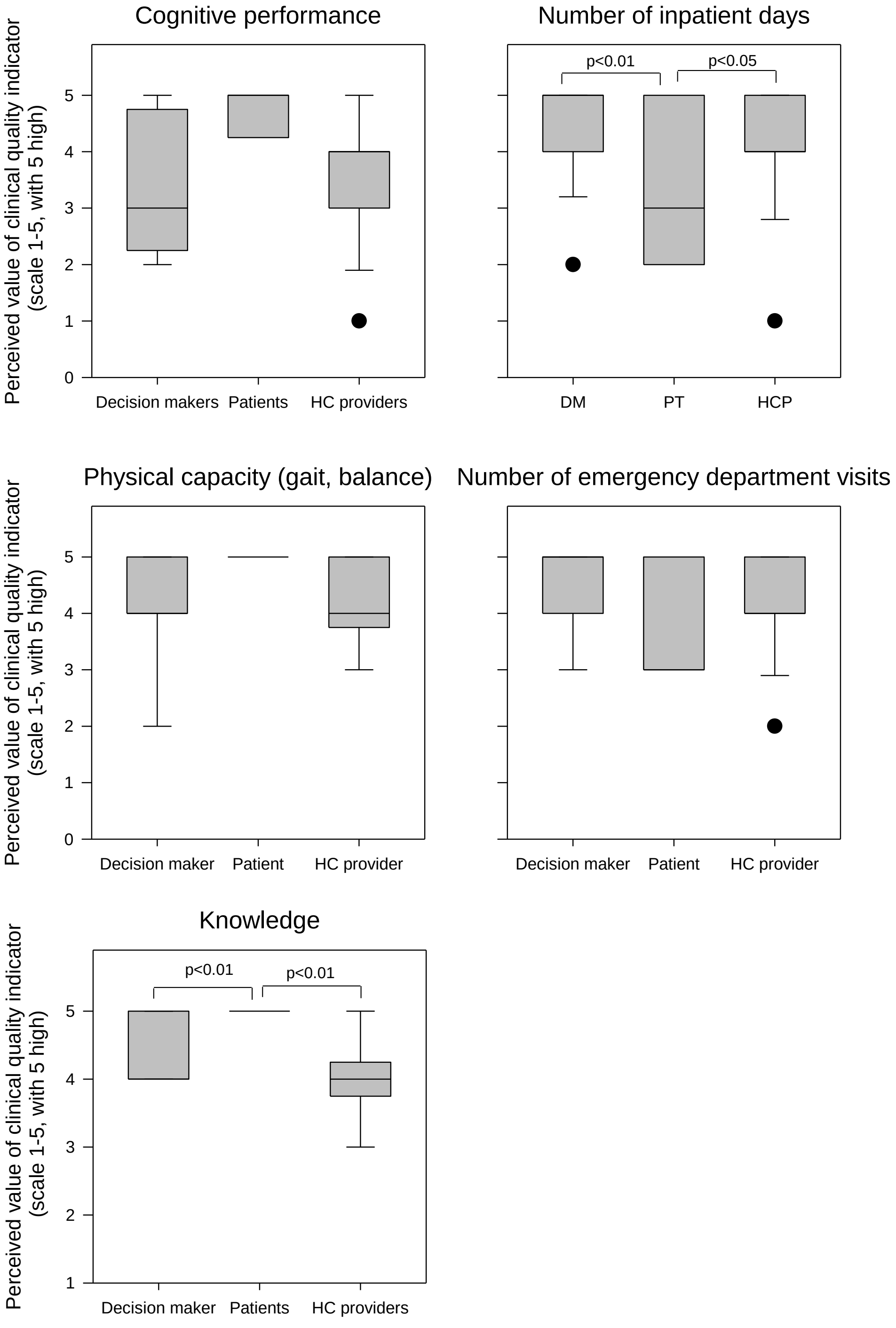

Supplement: Supplementary file 3 — Additional file 3: Distribution of participant perceptions of the importance of the five clinical quality indicators for which significant differences between participants were measured (median; box: 25th, 75th percentiles, whiskers: 10th, 90th percentiles; dots: outliers) [file 12877_2022_2843_MOESM3_ESM.pdf]
